# Task-sharing interventions for cardiovascular risk reduction and lipid outcomes in low- and middle-income countries: A systematic review and meta-analysis

**DOI:** 10.1016/j.jacl.2018.02.008

**Published:** 2018

**Authors:** T.N. Anand, Linju M. Joseph, A.V. Geetha, Joyita Chowdhury, Dorairaj Prabhakaran, Panniyammakal Jeemon

**Affiliations:** a Public Health Foundation of India, New Delhi, India; b Centre for Chronic Disease Control, New Delhi, India; c Sree Chitra Tirunal Institute for Medical Sciences and Technology, Trivandrum, Kerala, India

**Keywords:** Cardiovascular, Dyslipidemia, LDL-cholesterol, Task shifting, Task sharing

## Abstract

**Background:**

One of the potential strategies to improve health care delivery in understaffed low- and middle-income countries (LMICs) is task sharing, where specific tasks are transferred from more qualified health care cadre to a lesser trained cadre. Dyslipidemia is a major risk factor for cardiovascular disease but often it is not managed appropriately.

**Objective:**

We conducted a systematic review with the objective to identify and evaluate the effect of task sharing interventions on dyslipidemia in LMICs.

**Methods:**

Published studies (randomized controlled trials and observational studies) were identified via electronic databases such as PubMed, Embase, Cochrane Library, PsycINFO, and CINAHL. We searched the databases from inception to September 2016 and updated till 30 June 2017, using search terms related to task shifting, and cardiovascular disease prevention in LMICs. All eligible studies were summarized narratively, and potential studies were grouped for meta-analysis.

**Results:**

Although our search yielded 2938 records initially and another 1628 in the updated search, only 15 studies met the eligibility criteria. Most of the studies targeted lifestyle modification and care coordination by involving nurses or allied health workers. Eight randomized controlled trials were included in the meta-analysis. Task sharing intervention were effective in lowering low-density lipoprotein cholesterol (−6.90 mg/dL; 95% CI −11.81 to −1.99) and total cholesterol (−9.44 mg/dL; 95% CI −17.94 to −0.93) levels with modest effect size. However, there were no major differences in high-density lipoprotein cholesterol (−0.29 mg/dL; 95% CI −0.88 to 1.47) and triglycerides (−14.31 mg/dL; 95% CI −33.32 to 4.69). The overall quality of evidence based on Grading of Recommendations Assessment, Development and Evaluation was either “low” or “very low”.

**Conclusion:**

Available data are not adequate to make recommendations on the role of task sharing strategies for the management of dyslipidemia in LMICs. However, the studies conducted in LMICs demonstrate the potential use of this strategy especially in terms of reduction in low-density lipoprotein cholesterol and total cholesterol levels. Our review calls for the need of well-designed and large-scale studies to demonstrate the effect of task-sharing strategy on lipid management in LMICs.

## Highlights

• Task sharing is a potential health system support strategy for understaffed low- and middle-income countries.
• Task sharing interventions show moderate effect in lowering low-density lipoprotein cholesterol.
• Insufficient evidence to make recommendations for wider adoption of task sharing.

## Introduction

Consequent to epidemiologic transition, and population ageing, low- and middle-income countries (LMICs) are battling a double burden of disease. For example, LMICs are experiencing a rapid increase in noncommunicable diseases (NCDs), on top of the existing burden of communicable diseases, maternal health conditions, and nutritional disorders.^1^ The population size of LMIC is huge, and therefore, nearly 80% of the total 40 million deaths attributable to NCDs in absolute terms occur in these countries.^2^ Cardiovascular diseases (CVDs) are the leading contributor to NCD mortality and morbidity in LMICs. Largely CVD comprises of heart attack (myocardial infarction), angina, and stroke. The principal risk factors contributing to CVD are unhealthy diets, physical inactivity, exposure to tobacco smoke, and harmful alcohol consumption. These risk factors may lead to intermediate-level risk factors such as obesity, elevated levels of blood pressure, blood glucose, and blood lipids.^3^

Elevated blood lipids along with other risk factors are linked to CVD events, and the risk operates across the range of lipid profile, with a moderate reduction at the population level resulting in huge gain in terms of averted mortality and morbidity.^4^ It has been estimated that, a 10% reduction in serum cholesterol in men aged 40 years is associated with a 50% reduction in heart disease within 5 years; the same serum cholesterol reduction for men aged 70 years may result in an average 20% reduction over the next 5 years.^5^

Shifting the population distribution of serum cholesterol toward the left of the distribution curve, even marginally, requires a combination of population-wide primary prevention efforts addressing multiple risk factors and high-risk secondary prevention strategies. However, the health workforce available in LMICs to address the dual burden of both communicable diseases and NCDs are very limited. For instance, on an average, there are 0·3 doctors available for every 1000 population in low income countries, 1·2 doctors available for every 1000 population in LMICs, and 2 doctors available for 1000 population in upper middle-income countries, respectively.^6^ In resource constrained settings with fewer physicians, it would be a desirable choice in using the existing nonphysician health care workers (NPHW) for the prevention and control of NCDs.

Task shifting or task sharing or task delegation or skills substitution are all referred to as the process of engaging NPHW in prevention and control of NCDs in the context of LMICs.^7^ However, it is not clear whether these strategies would be effective in cholesterol reduction in individuals and communities. Previous studies demonstrate that task-sharing strategies for hypertension and diabetes management are both viable^8^, ^9^ and cost-effective^10^ options in LMICs. The primary focus of the current review is to identify and understand the various task-sharing interventions used in the management of dyslipidemia in LMICs, and their cumulative effect on total cholesterol (TC), low-density lipoprotein cholesterol (LDL-c), high-density lipoprotein cholesterol (HDL-c), and triglycerides (TGs). We aimed to generate evidence to support informed policy decisions on the role of task-sharing strategies for the management of dyslipidemia in LMICs and provide recommendations on the need for future research.

## Methods

### Definitions

Task sharing is defined as the rational redistribution of tasks to an existing or new cadre of health workers with either less training in general or lack of disease-/skill-specific training. It involves sharing the delivery of the task from professionals to health workers with fewer qualifications or creating a new workforce with specific training for a specific task.^11^ Health professionals working together in teams to deliver a task that they may not have undertaken previously is also considered as task sharing.^5^

We searched the published literature for studies (randomized controlled trials [RCTs], observational studies, and before and after studies) conducted in LMICs that included a task-shared intervention, delivered by nurses or NPHW in primary health centers or hospitals. Outcome measures included were TC, LDL-c, HDL-c, and TGs. Only studies in adult participants were considered.

### Exclusions

Studies with patient's knowledge, attitudes, or intentions as outcome variables without measuring any of the relevant lipid outcomes were excluded. Interventions that involve only peer groups were excluded as they would be more likely to be informal support. In addition to these, task-sharing activities that are exclusive to traditional healers and those with just the promotion of self-care management or informal care giver health education were excluded in this review. Studies that do not report a change in TC or LDL-c were also excluded.

## Search strategy

A systematic literature search in 5 bibliographic databases (PubMed, Cochrane Library, CINAHL, Embase and PsycINFO) was conducted from inception to September 2016. We adapted a search strategy from a previous review in 2014,^8^ initially modified it (Appendix 1, online supplement) for PubMed, and subsequently modified for other databases. Comprehensive search was carried out for studies performed in LMICs classified according to the World Bank Lending Group,^12^ and was updated again in PubMed by adding the search term “Sub-Saharan Africa”. No limits on language or publication year were applied during the literature search. The keywords used were categorized into 3: for finding disease, for identifying task-shifted intervention, and for finding studies carried out in LMICs. The keywords were combined using appropriate Boolean operators such as “cardiovascular disease” OR “hyperlipidemia” OR “diabetes” OR “heart failure” AND “task” OR “shifted” OR “shared” OR “nonphysician health care worker” OR “community care worker” AND “developing countries” OR “low-income countries” OR “resource poor”. Bibliographies of relevant studies were searched, and cross-referenced to identify any additional studies relevant for inclusion.

### Data collection and analysis

#### Selection of studies

Two investigators (T.N.A. and J.L.M.) independently reviewed titles and abstract of all relevant articles identified. Those studies that appeared to be on task-sharing interventions for CVD prevention or management in LMIC were selected for full-text review. T.N.A. and J.L.M. further reviewed these full-text articles independently to identify studies on lipid management. A third investigator (P.J.) served as a tiebreaker, independently reviewing articles to resolve the disagreement between the other 2 investigators. The Preferred Reporting Items for Systematic Reviews and Meta-Analyses (PRISMA) guidelines was followed to report the review process.^13^

### Data management and statistical analysis

Data extraction was carried out by 2 investigators (T.N.A. and J.L.M.). Queries regarding data extraction were resolved mutually by returning to the original article and reviewing the data. Studies were grouped in terms of countries of focus, the cadres discussed, the disease focused, and interventions used for control of lipids. The interventions were summarized narratively.

After data extraction, task-sharing interventions in all studies were summarized using narrative synthesis. Eligible RCTs were grouped to perform a meta-analysis. The quality of individual studies was appraised using National Heart, Lung, and Blood Institute^14^ (NHLBI) quality assessment scale for nonrandomized before and after studies. This tool contains 12 items on the risk of potential for selection bias, information bias, measurement bias, and confounding. Cochrane Risk of Bias Tool was used for assessing the quality of RCTs. This tool examined randomization procedure, allocation concealment, blinding of outcome assessors, incomplete outcome data, and selective outcome reporting. For the cluster trials, we evaluated recruitment bias, baseline imbalances, loss of clusters, incorrect analysis, and comparability with individual RCT as outlined in the Cochrane Handbook for Systematic Reviews of Interventions.^15^ Two independent reviewers (T.N.A. and J.L.M.) assessed risk of bias, differences between reviewers were resolved by discussion with a third reviewer (P.J.), and a consensus was reached.

Meta-analyses of eligible RCTs were conducted on each outcome of interest independently. Results were synthesized based on outcome measures of the included studies. We used R version 3.3.2 with “metaphor and meta” packages for meta-analysis. Mean difference (MD) in cholesterol levels between the intervention, and control arm were estimated. Cholesterol values expressed in mmol/L was converted to mg/dL. Overall effects were calculated by combining the individual study effects using random effects model, and estimates were reported with 95% CI. To detect heterogeneity, we used Q statistics and I^2^ values. Appropriate subgroup analyses were also conducted. Funnel plots and Egger's regression test for funnel plot asymmetry were performed.

We assessed the quality of the evidence for each outcome across studies included in the meta-analyses according to the Grading of Recommendations Assessment, Development and Evaluation (GRADE) approach.^16^ The quality of evidence was rated as high, moderate, low, or very low after determination of within-study risk of bias (methodological quality), directness of evidence, heterogeneity, the precision of effect estimates, and risk of publication bias.

## Results

**Figure 1:**
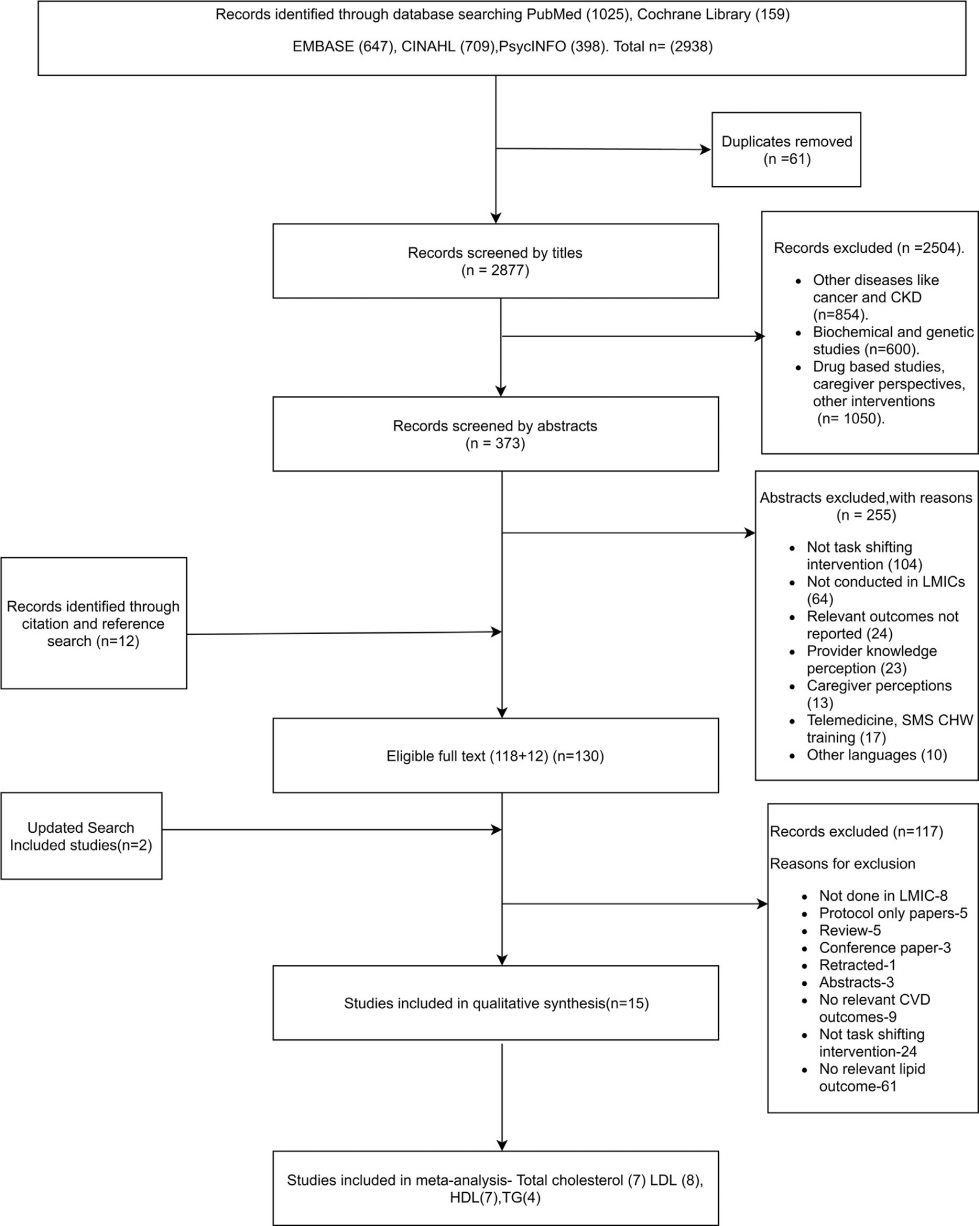
Flow diagram showing literature search and final articles included in the review. SMS, short messaging service; CHW, community health worker; CKD, chronic kidney disease; LMIC, low- and middle-income countries; LDL, low-density lipoprotein; HDL, high-density lipoprotein; CVD, cardiovascular disease.

Our search yielded a total of 2938 potential citations from PubMed (n = 1025), Cochrane Library (n = 159), Embase (n = 647), CINAHL (n = 709), and PsycINFO (n = 398). Duplicates were removed (n = 61). Title screening initially removed 2504 articles. All the remaining 373 abstracts were analyzed for eligibility, and 255 abstracts were excluded. Additional 12 articles from hand search (citation and references) were obtained. In total, 130 articles were initially included for full-text reading, and 117 of them were discarded based on the full-text review. The reasons for exclusion of articles during full-text review were nonavailability of outcomes of cardiovascular risk reduction (n = 9), not being done in LMICs (n = 8), interventions without task-sharing strategy (n = 24), absence of outcomes related to lipids management (n = 61), and others such as conference papers, abstracts, and review (n = 15). We further updated our search in PubMed till 30 June 2017 (n = 1628). 163 abstracts were reviewed and 4 full texts were found and finally 2 studies were added. Finally, 15 articles were included in the detailed review, and presented in the PRISMA flow diagram.^13^ (Fig. 1).

### General characteristics of included studies

Of the 15 studies included (Table 1), 1 study was a cluster randomized trial,^23^ 10 individual RCTs,^17^, ^18^, ^19^, ^20^, ^21^, ^22^, ^24^, ^25^, ^26^, ^27^ and another 4 were before and after studies.^28^, ^29^, ^30^, ^31^ There were 2 studies each from South Africa,^23^, ^24^ Brazil,^17^, ^22^ and China,^18^, ^27^ 1 each from India,^25^ Malaysia,^21^ Russia,^20^ and Turkey.^19^ One study was carried out as a multicenter trial in India and Pakistan.^26^ The before and after studies were carried out 2 each, in Iran,^28^, ^31^ and 1 each from Mexico^29^ and Thailand.^30^ The earliest study in the review was reported in 2005,^17^ and the most recent study was reported in 2017.^27^ Participant follow-up ranged from 8 weeks^28^ to 36 months.^26^ In total, 6 of 15 studies reported a follow-up of 1 year. Four studies were carried out in the tertiary hospital setting, 1 each in a private clinic and diabetic clinic, and other studies in community health centers or primary care practices.

**Table 1:** Characteristics of studies included in this review

Author, country, year reported	Study design	Disease type	Task shifted to/shared with	Sample, intervention control	Setting, duration	Intervention and control group	Outcomes measured	Main lipid results from studies
Sartorelli *et al* Brazil, 2005^17^	RCT	High-risk group such as overweight or obese adults and relatives of patients with type II diabetes mellitus	Nutritionist	104, I = 51, C=53	Primary health centre, 12 mo	I: Individualized dietary counseling C: Routine care	Changes in CVD risk factors (blood pressure, lipids, diabetes, obesity)	At the 6-mo follow-up, significant difference in total cholesterol (−12·3% vs 0·2%) and (LDL-c) (−15·5% vs +4·0%) (*P*, .05). At 12-mo follow-up, the reduction in LDL-c levels (−13·3%) in the intervention group was significant when compared with baseline.
Jiang *et al* China, 2006^18^	RCT	Coronary heart disease	Nurses	167, I = 83 C = 84	Tertiary medical centre and home, 12 wk	I: Hospital-based patient/family education and home-based cardiac rehabilitation. C: Routine care	1. Lifestyle parameters: smoking cessation, walking performance, step II diet adherence 2. Clinical (serum lipids, body weight and blood pressure)	The intervention was successful in reducing TG, TC, and LDL-c at both 3 mo (*P* < .01, *P* < .001) and 6 mo (*P* < .05, *P* < .001) but no difference was observed for HDL.
Mollaoğlu *et al* Turkey, 2009^19^	RCT	Diabetes	Nurses	50, I = 25 C = 25	Hospital and home	I: Predischarge health education for metabolic control and follow-up at home. C: Routine care	Clinical parameters: HbA1c, FBS PPBS, urine glucose, and cholesterol (total cholesterol, TG, HDL-c, and LDL-c).	Total cholesterol and LDL-c were found to have a significant difference after nurse education.
Andryukhin *et al* Russia, 2010^20^	RCT	Heart failure	Nurses	85, I = 44 C = 41	GP Practice and home, 12 mo	I: Educational programme for patients with heart failure C: Patients managed with Russian National guidelines.	1. Lifestyle parameters: 6-min walking test New York Heart Association Class of CHF, BMI, WC 2. Clinical parameters of blood plasma levels of fasting blood glucose, total cholesterol, LDL-c, CRP, (high sensitivity method) and NT-pro BNP	Significant improvement in total cholesterol, low-density lipoprotein, after 6 mo for intervention group. Total cholesterol. mmol/L, Median, IQR Baseline C: 5.53 (5.31–6.13) I: 6.10 (5.72–6.42) At 6 mo C: 5.60 (5.43–6.18) I: 5.30 (5.28–6.05) LDL-c mmol/L, median, IQR Baseline C: 3.57 (3.34–4.03) I: 3.795 (3.58–4.28) At 6 mo C:3.72 (3.62–4.28) I: 3.505 (3.34–4.04)
Selvaraj *et al* Malaysia, 2012^21^	RCT	Dyslipidemia	Nurse educators	297, I = 149 C = 148	Primary care practices, 36 wk	I: physician and nurse educator COACH Programme received biweekly telephone follow-up by trained nurse educators and reinforcement for medication adherence. C: Routine care(PCP alone)	Change in HbA1c at 6 mo. Other outcomes were changes in other clinical outcomes (BMI, blood pressure and blood lipids), HbA1c and dietary behaviors at 12 mo.	Intervention group showed better improvements in both LDL-c and TC levels when patients were co-managed by nurse educators but was not statistically significant.
Saffi *et al* Brazil, 2014^22^	RCT	Coronary heart diseases	Nurses	74, I = 38 C = 36	Tertiary referral hospital, 12 mo	I: individual counseling sessions and telephone follow-up C: Routine care	Reduction of estimated 10-y CVD risk (Framingham Risk Score). Clinical (lipid profile, blood glucose, HbA1c) and anthropometric parameters (weight, BMI, WC, WHR), BP, capillary blood glucose measurements, and adherence.	Total cholesterol mg/dL (mean and SD) at 1 y 175 ± 53 vs 173 ± 33, *P* = .24, LDL-c mg/dL (mean and SD) at 1 y 99 ± 47 vs 96 ± 25, *P* = .03, HDL-c mg/dL (mean and SD) at 1 y −43 ± 8 vs 42 ± 9, *P* = .06, TG mg/dL (median and IQR) at 1 y −145 (9–224) vs 164 (102–223), *P* = .13
Mash R J *et al* South Africa, 2014^23^	Cluster RCT	Diabetes	Health promoter	1570 I = 860 C = 710	Community health centers, 12 mo	I: group diabetes education led by a health promoter. C: Routine care	1. Improved diabetes self-care activities, 5% weight loss, and a 1% reduction in HbA1c level. 2. Secondary outcomes were improved diabetes-specific self-efficacy, locus of control, mean blood pressure, mean weight loss, mean waist circumference, mean HbA1c and mean total cholesterol levels, and quality of life	No significant improvement was found. Total cholesterol, mmol/mean difference between control and intervention group −0.13 (−0.27 to 0.01) (weighted means as per analysis model), *P* = .066
Muchiri *et al* South Africa, 2015^24^	RCT	Diabetes	Dietitian	82 I = 41 C = 41	Community health centers, 12 mo	I: Nutrition Education programme C: group participants received education materials (pamphlet and wall/fridge poster) and usual medical care.	Change in HbA1c, BMI, lipid profile, blood pressure and intakes of macronutrients, vegetables, and fruits	No significant results for lipid profile. Post Intervention values: Cholesterol (157.0 [40.2] vs 166.9 [48.4]; *P* = .184), LDL-c (81.0 [20.6] vs 87.3 [29.9]; *P* = .191), HDL-c (42.0 [11.4] vs 38.2 [6.5]; *P* = .042)
Xavier *et al* India, 2016^25^	RCT	Acute Coronary Syndrome	Community health workers (CHW)	806, I = 405, C = 401	Tertiary hospital and home, 12 mo	I: community health worker–based intervention for adherence to drugs and lifestyle change after acute coronary syndrome. Four in-hospital and 2 home visits for medication adherence C: Routine care	Adherence to proven secondary prevention drugs. Others were lifestyle change, including diet, exercise, and tobacco and alcohol use, which were assessed by different scores and clinical risk markers (blood pressure, heart rate, body weight, BMI, and lipids).	At 1 y, cholesterol (157.0 [40.2] vs 166.9 [48.4]; *P* = .184), LDL-c (81.0 [20.6] vs 87.3 [29.9]; *P* = .191), HDL-c (42.0 [11.4] vs 38.2 [6.5]; *P* = .042), were lower in the intervention group than in the control group but not statistically significant.
Ali *et al* India and Pakistan, 2016^26^	RCT	Diabetes	Nonphysician care coordinators *nonphysicians with training in allied health fields (such as dietetics or social work), at least 6 mo of health care experience, and good organizational and basic computing skills.	1146, I = 575 C = 571	Outpatient diabetic clinics, 36 mo	I: Multicomponent Quality Improvement strategy comprising nonphysician care coordinators and decision-support electronic health records. C: Routine care	Primary outcome was the proportion of patients from each group achieving an HbA1c level less than 7% plus a BP less than 130/80 mm Hg and/or an LDL-c- level less than 2·59 mmol/L (<100 mg/dL) (<1.81 mmol/L [<70 mg/dL] for patients with a history of CVD).	Compared with usual care, intervention participants attained LDL-c level (−7.86 mg/dL [CI, −10.90 to −4.81 mg/dL]). Initial TG > 1·69 mmol/L (150 mg/dL) I = 67 (63) C = 63 (59) not significant; Final TG > 1·69 mmol/L (150 mg/dL) I = 16 (14) C = 29 (27) *P* < .05. Initial LDL-c > 2.58 mmol/L (100 mg/dL) I = 74 (69%) C = 70 (65%) not significant Final LDL-c > 2.58 mmol/L (100 mg/dL) 14 (18%) 27 (25%) *P* < .04
Zhang *et al* China, 2017^27^	RCT	Coronary artery disease	Nurses	199 I = 100 C = 99	General hospital 7 mo	Nurse led transitional care vs routine care	1. C: SBP, DBP, FBS, TC, triglyceride, HDL-c, LDL-c and BMI. 2. Knowledge scale for CAD 3. SF-36	The experimental group showed significant clinical outcome SBP, t = 5.762, *P* = .000; DBP, t = 4.250, *P* = .000; FBS, t = 2.249, *P* = .027; t = 4.362, *P* = .000; triglyceride, t = 3.147, *P* = .002, LDL-c, t = 2.399, *P* = .018; and BMI, t = 3.166, *P* = .002 and higher knowledge scores for coronary artery disease
Pishdad *et al* Iran, 2008^28^	Before and after	Diabetes	Nurses	214, I = 107 C = 107	Private Endocrinology Clinic, 12 mo	NADC model vs routine care	HbA1c, TG, LDL-c, cholesterol, duration of patient's visit and net clinic's income for patients under NADC were compared with those of usual care.	Significantly smaller proportions of patients had triglyceride levels of > 1.69 mmol/L and LDL-c of > 2.58 mmol/L (both *P* < .05) in the nurse-assisted group.
Denman *et al* Mexico, 2012^29^	Before and after	Low-income participants with high risk for developing CVD	CHW	166	Community health centers, 13 wk	Health education classes by CHW for heart healthy lifestyle vs Pasos Adelante outcomes	Anthropometric waist and hip circumference, weight for calculating BMI (kg/m2); clinical biomarkers fasting blood glucose, HDL-c, LDL-c-, total cholesterol, and triglycerides and lifestyle questionnaire	Significant changes from baseline to conclusion in LDL-c (7.93 [95% CI, 1.02–14.8] mg/dL), and triglycerides (−26.4 [95% CI, −40.4 to −12.4] mg/dL).
Navicharern *et al*, Thailand, 2009^30^	Before and after	Diabetes	Nurse	40, I = 20 C = 20	Two red cross health stations, 12 wk	Nurse coaching vs routine care	HbA1c, blood pressure and LDL-c-testing, and satisfaction with nursing intervention questionnaire	No significant results for LDL-c.
Kamran *et al* 2016, Iran^31^	Before and after	Individuals with hypertension	Health promotion specialist	138, I = 68 C = 70	Rural health center, 6 mo	Nutritional advice by health promotion specialist vs routine care with instructional booklets	Mean change in total fat intake, saturated fat, dietary cholesterol and weight. Clinical outcomes such as HDL-c, TC < LDL-c, SBP and DBP.	Intervention group had significant decrease in weight, dietary fat, LDL-c and, TC, SBP and DBP compared with the control group (*P* < .001).

RCT, randomized controlled trial; I, intervention group; C, control group; LDL, low-density lipoprotein; LDL-c, low density lipoprotein cholesterol; HDL, high-density lipoprotein; TC, total cholesterol; TG, triglycerides; HbA1c, glycosylated hemoglobin; CVD, cardiovascular disease; BMI, body mass index; WC, waist circumference; WHR, waist-to-hip ratio; IQR, interquartile range; BP, blood pressure; FBS, fasting blood sugar; PPBS, post prandial blood sugar; COACH, counseling and advisory care for health; PCP, primary care physician; CHF, congestive heart failure; GP, general physician; NT-pro BNP, N-terminal pro-brain natriuretic peptide; cRP, C reactive protein; NADC, nurse-assisted diabetes care; SD, standard deviation.

Mean age reported in the studies ranged from 42 to 67 years, and the proportion of female participants ranged from 26% to 98%. Participants included in the studies reported varying past medical histories. Most studies recruited participants with diabetes or CVD. One study exclusively included dyslipidemia participants.^21^ Other studies included obese participants,^17^ individuals with hypertension,^31^ heart failure,^20^ and acute coronary syndromes.^25^

### Task-sharing interventions in included studies

#### Findings from trial

Most of the studies (n = 6) implemented task-sharing interventions involving nurses (Table 1). In one study each tasks were delegated to dietitian, health promoter, and care coordinator. In addition, 2 studies employed other NPHW to deliver the intervention. The type of task-sharing interventions used markedly varied across studies. Major task-sharing interventions identified were lifestyle modification health education and follow-up using the telephone or home visits.

Three studies^17^, ^23^, ^24^ used lifestyle modification health education as their main intervention. Of the 3 studies, 2 studies^17^, ^24^ focused on counseling by a dietitian on diet and physical activity as their main intervention. Another study used a nurse to impart lifestyle modification education^23^ and adherence management along with diet and physical activity counseling.

Eight trials^18^, ^19^, ^20^, ^21^, ^22^, ^25^, ^26^, ^27^ used both lifestyle modification and follow-up. Xavier et al^25^ employed NPHW to impart lifestyle modification education for patients discharged from hospital after acute coronary syndrome. Ali et al^26^ employed technology enabled coordinators with decision support system, and they acted as a link between diabetic patients and treating physicians. Two other studies^18^, ^26^ further stressed the importance of self-monitoring for glucose. Two studies^19^, ^21^ used nurses for follow-up and counseling. Four trials^19^, ^20^, ^23^, ^24^ delivered group health education, and other 4 studies^17^, ^18^, ^22^, ^25^ used individualized face-to-face health education. Four studies used telephone^20^, ^21^, ^22^, ^26^ follow-up for the participants, 2 studies^19^, ^25^ used home visits as follow-up, and Jiang et al^18^ used both home visit and telephone follow-up alternatively. Most studies reported a training component for the cadre delivering the interventions, although they differed with respect to content, duration, and refresher training availability. The outcome measures and results of the interventions are presented in Table 1.

#### Findings from nonrandomized before and after studies

The participants were patients with diabetes visiting a diabetic clinic in Iran,^28^ 2 health stations in Bangkok,^30^ individuals with hypertension^31^ referred to rural health center and low-income residents of an urban area of Northern Mexico.^29^ Of the 4 studies, 2 used nurses^28^, ^30^ for delivering interventions. The other 2 employed health promotion^31^ specialists and NPHW^29^ . Three studies^28^, ^29^, ^31^ emphasized on lifestyle modification health education. The study with NPHW, delivered health education focused on diet and physical activity, whereas the study with nurses focused on adherence management along with diet and physical activity. Kamran et al^31^ described how health promotion specialist delivered nutritional education based on Dietary Approach to Stop Hypertension diet. Denman et al^29^ conducted physical activity sessions 1 to 3 times per week. One study^30^ had employed nurses for health education and follow-up. Two studies^28^, ^30^ used individual health education, whereas the other 2 studies^29^, ^31^ used group health education. Navicharern et al^30^ used telephone follow-up for their participants. One study compared the same participants before and after intervention without a control group,^29^ and the other 3 studies had a control group, which received usual care.^28^, ^30^, ^31^ The outcome measures are presented in Table 1. Kamran et al found that at 6 months after intervention, LDL-c and TC decreased significantly in the intervention group compared with the control group (*P* < .001). Navicharern et al^30^ reported no differences in the levels of LDL-c for participants after the intervention. At the end of 3-month follow-up, Denman^29^ found significant differences from baseline for TC (14.2 mg/dL [95% CI, 6.6–21.8]), HDL-c (−11.1 mg/dL [95% C, −14.1 to −8.1]), and LDL-c (21.6 mg/dL [95% CI, 14.0–29.2]). At the end of 6-month follow-up, Pishdad^28^ reported reduction in the proportion of patients with TG concentrations of > 1·69 mmol/L (150 mg/dL) from 63% to 14% (nurse assisted) vs 59% to 27% (usual care) (*P* < .05). In addition, for LDL-c concentrations of > 2.58 mmol/L (100 mg/dL), proportion of patients decreased from 69% to 18% (nurse assisted) vs 65% to 25% (usual care) (*P* < .04).

### Quality of included studies

Overall, some risk of bias was evident in all the studies included in the review (Table S1, online supplement). Eight RCTs^18^, ^20^, ^21^, ^22^, ^23^, ^24^, ^25^, ^26^, ^27^ clearly described a random sequence generation method (eg, computer generated random number table) and were deemed to be at low risk. Two RCTs^17^, ^19^ did not report the method of randomization and hence marked as at unclear risk. Allocation concealment was clearly specified in 5 studies,^18^, ^19^, ^20^, ^24^, ^25^ and they were assigned low risk. One RCT^22^ reported no allocation concealment, and in another 4,^16^, ^17^, ^21^, ^23^ the risk was unclear. Overall, most RCTs^17^, ^20^, ^21^, ^22^, ^23^, ^24^, ^26^, ^27^ were at risk of performance bias. Some of the studies^17^, ^19^, ^21^ did not clearly mention about blinding of outcome assessors. Most of the studies had a relatively lower loss to follow-up and used intention to treat analysis. However, the attrition rate was more than 50% in the cluster RCT.^23^ Low risk of bias for selective reporting was observed in 4 RCTs,^21^, ^22^, ^23^, ^26^ whereas it was difficult to report it based on available information in the remaining studies.

Using the National Heart, Lung, and Blood Institute scale, the before and after studies were assessed for methodological quality (Table S2, online supplement). All the before and after studies reported study objectives, although 1 study^29^ did not clearly mention the eligibility criteria. Three^29^, ^30^, ^31^ of 4 studies did report on sample size calculation. All 4 studies^28^, ^29^, ^30^, ^31^ described the intervention and outcome measures. Outcome assessors were not blinded in any reported studies. All 4 studies were relatively of shorter duration, and the loss to follow-up was <20%.

### Effects of intervention on outcomes

#### Low-density lipoprotein cholesterol

**Figure 2:**
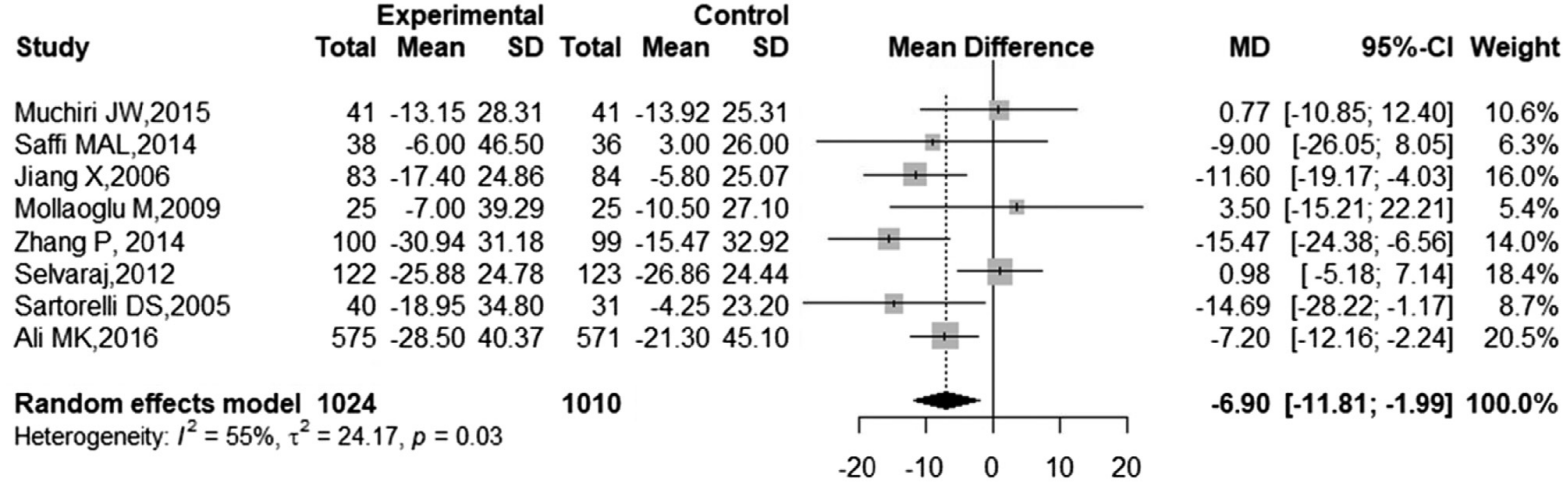
Forest plot showing changes in low-density lipoprotein (LDL) levels; comparison of task-sharing interventions with usual care. MD, mean difference; SD, standard deviation; CI, confidence intervals.

Eight RCTs were included in the meta-analysis of the effect of task-shifting interventions on LDL-c levels. In total, 2034 study participants (intervention group [n = 1024], control group [n = 1010]) were included in the final meta-analysis. The intervention period ranged from 2 to 24 months. The pooled MD based on random effects model was −6.90 mg/dL; (95% CI −11.81 to −1.99; *P* = .03; Fig. 2). The chi-square test showed significant heterogeneity (χ^2^ = 15.46, *P* = .03, I^2^ = 54.7%). Test for funnel plot asymmetry was not significant (*P* = .86) (Fig. S1). The overall quality of evidence based on GRADE was however “low” (Table 2).

**Table 2:** Summary of findings for main outcomes

Task sharing compared with usual care for dyslipidemia						
Patient or population: Individuals at risk of developing CVD or CVD related complications. Setting: low and middle income Intervention: Task sharing Comparison: Usual care						
Outcomes	Anticipated absolute effects∗ (95% CI)			No of participants (studies)	Quality of the evidence (GRADE)	Comments
	Risk with usual care	Risk with task shifting	Relative effect (95% CI)			
LDL follow-up: range 2 mo to 24 mo	The mean low-density lipid was −18.47 mg/dL	The mean low-density lipoprotein in the intervention group was 6.90 mg/dL lower (11.81 lower to 1.99 lower)	–	2034 (8 RCTs)	⊕⊕◯◯ Low	
HDL follow-up: range 2 to 12 mo	The mean high-density lipid was 0.37 mg/dL	The mean high-density lipoprotein in the intervention group was 0.29 mg/dL higher (1.12 lower to 1.94 higher)	–	888 (7 RCTs)	⊕⊕◯◯ Low	
TC follow-up: range 2 to 12 mo	The mean total Cholesterol was −16.99 mg/dL	The mean total cholesterol in the intervention group was 9.44 mg/dL lower (17.94 lower to 0.93 lower)	–	888 (7 RCTs)	⊕⊕◯◯ Low†‡§	
TG follow-up: range 2 to 12 mo	The mean triglycerides were −18.12 mg/dL	The mean triglycerides in the intervention group was 14.31 mg/dL lower (33.32 lower to 4.69 higher)	–	487 (4 RCTs)	⊕◯◯◯ Very lowǁ¶#	

CI, confidence interval; MD, mean difference; LDL, low-density lipoprotein; HDL, high-density lipoprotein; TC, total cholesterol; TG, triglycerides; CVD, cardiovascular disease; RCT, randomized controlled trial.

GRADE Working Group grades of evidence.

High quality: We are very confident that the true effect lies close to that of the estimate of the effect.

Moderate quality: We are moderately confident in the effect estimate: The true effect is likely to be close to the estimate of the effect, but there is a possibility that it is substantially different.

Low quality: Our confidence in the effect estimate is limited: The true effect may be substantially different from the estimate of the effect.

Very low quality: We have very little confidence in the effect estimate: The true effect is likely to be substantially different from the estimate of effect.

∗ The risk in the intervention group (and its 95% confidence interval) is based on the assumed risk in the comparison group and the relative effect of the intervention (and its 95% CI).

† High risk of bias.

‡ Wide variation in study population, intervention and task shifting strategies.

§ Few study participants with wider CI.

ǁ High risk of bias characterized by no Random Sequence Generation, Poor outcome assessment.

¶ Variations in interventions and study population.

# Fewer study participants with wide variation in features.

##### Subgroup analysis (LDL-c)

In individuals with diabetes, the pooled estimate was −4.46 mg/dL (95% CI: −10.40 to 1.48) (Fig. S2), whereas it was −12.79 mg/dL (95% CI: −18.26 to −7.32) in patients with coronary artery disease (CAD) (Fig. S2). Based on the task-sharing group, pooled estimate for nurses was −6.98 mg/dL (95% CI: −14.91 to 0.94), whereas it was −6.56 mg/dL (95% CI: −21.70 to 8.52) in studies involving dietitians (Fig. S3).

#### High-density lipoprotein cholesterol

**Figure 3:**
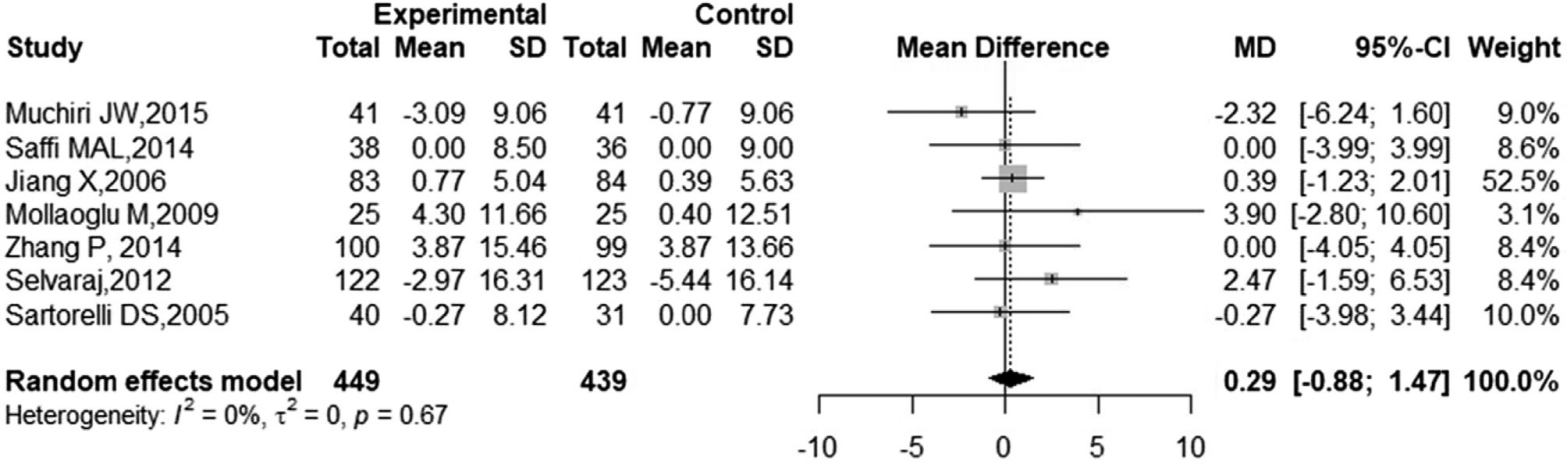
Forest plot showing changes in high-density lipoprotein (HDL) levels; comparison of task-sharing interventions with usual care. MD, mean difference; SD, standard deviation; CI, confidence intervals.

Seven RCTs were included in the meta-analysis of the effect of task-sharing interventions on HDL-c levels. A total of 888 study participants (intervention group [n = 449], control group [n = 439]) were included in this meta-analysis. Intervention period ranged from 2 to 12 months. The pooled estimate in the random effects model indicated no additional benefit in the intervention group in comparison with the usual care group (MD = −0.29 mg/dL; 95% CI −0.88 to 1.47; *P* = .62; Fig. 3). The chi-square test showed no heterogeneity (χ^2^ = 4.06, *P* = .66, I^2^ = 0.0%). Egger's regression test for funnel plot asymmetry showed no publication bias with *P* value .76 (Fig. S4). The overall quality of evidence based on GRADE was “low” (Table 2).

##### Subgroup analysis (HDL-c)

The pooled MD of HDL-c levels was 0.17 mg/dL (95% CI: −5.80 to 6.15; Fig. S5) and 0·29 mg/dL (95% CI: −1.12 to −1.70; Fig. S5) in patients with diabetes and CAD, respectively. Interventions with nurses resulted in an MD of 0.65 mg/dL (95% CI: −0.65 to 1.96; Fig. S6), whereas it was −1.24 mg/dL (95% CI: −3.93 to 1.46; Fig. S6) in studies involving dietitians.

#### Total cholesterol

**Figure 4:**
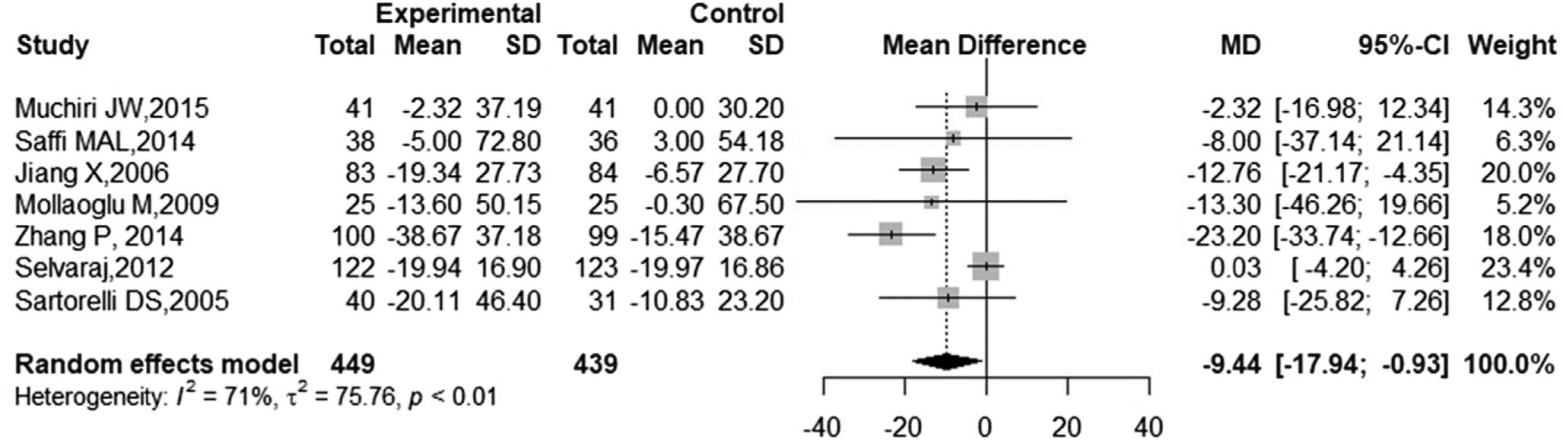
Forest plot showing changes in total cholesterol levels; comparison of task-sharing interventions with usual care. MD, mean difference; SD, standard deviation; CI, confidence intervals.

Seven RCTs were included in the meta-analysis of the effect of task-sharing interventions on TC levels. A total of 888 study participants (intervention group [n = 449], control group [n = 439]) were included in this meta-analysis. Intervention period ranged from 2 to 12 months. The pooled estimate in the random effects model differ between the intervention and control group (MD = −9.44; 95% CI −17.94 to −0.93; *P* = .01; Fig. 4). The chi-square test showed statistical heterogeneity (χ^2^ = 20.83, *P* = .00, I^2^ = 71.2%). There was no publication bias in the Egger's regression test (*P* = .23; Fig. S7). The overall quality of evidence based on GRADE was “low” (Table 2).

##### Subgroup analysis (TC)

Pooled estimates for the effect of interventions on TC levels in patients with diabetes and CAD were −4.13 mg/dL (95% CI: −17.53 to 9.26; Fig. S8) and −16·57 mg/dL (95% CI: −24.59 to −8.56; Fig. S8), respectively. Interventions involving nurses resulted in an MD of −11.07 mg/dL (95% CI: −22.50 to 0.36; Fig. S9), whereas it was −5.38 mg/dL (95% CI: −16.36 to 5.59; Fig. S9) in studies involving dietitians.

#### Triglycerides

**Figure 5:**
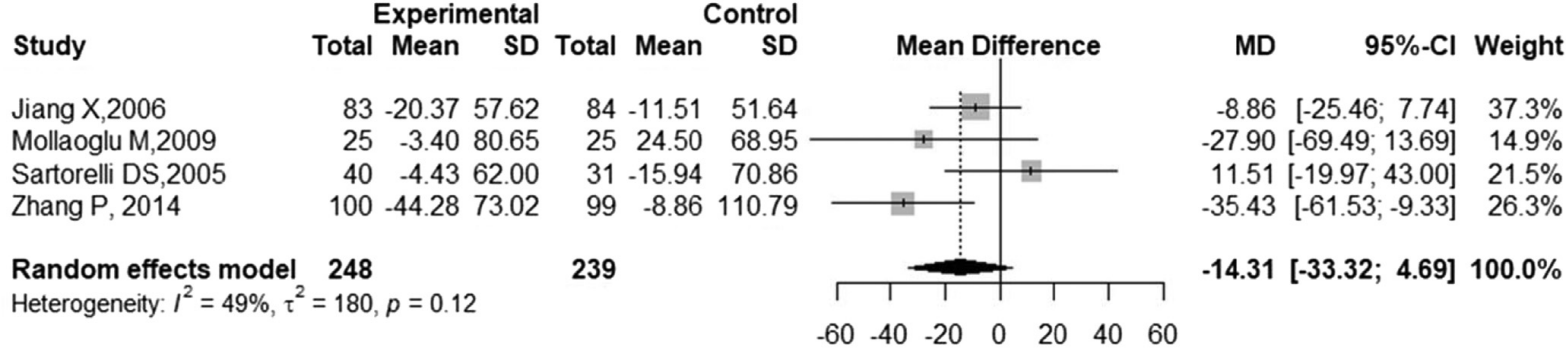
Forest plot showing changes in triglyceride levels; comparison of task-sharing interventions with usual care. MD, mean difference; SD, standard deviation; CI, confidence intervals.

Four RCTs were included in the meta-analysis of the effect of task-sharing interventions on TG levels. A total of 487 study participants (intervention group [n = 248], control group [n = 239]) were included in this meta-analysis. Intervention period ranged from 2 to 12 months. The pooled estimate, based on random effects model, did not differ between groups (MD = −14.31 mg/dL; 95% CI −33.32 to 4.69; *P* = .13; Fig. 5). The chi-square test showed no statistical heterogeneity (χ^2^ = 5.90, *P* = .11, I^2^ = 49.1%). There was no publication bias in the funnel plot asymmetry test (*P* = .81; Fig. S10). The overall quality of evidence based on GRADE was “very low”. (Table 2). Complete information of GRADE assessment is provided in supplementary file (Table S3, online supplement).

##### Subgroup analysis (TG)

Pooled estimate of intervention effect in patients with CAD was −20.16 mg/dL (95% CI: −45.90 to 5.59; Fig. S11). Pooled estimates for TG based on the interventions implemented by nurses was −20.42 mg/dL (95% CI: −38.80 to −2.03; Fig. S12).

Detailed PRISMA check list is provided in the Figure S4, online supplement.

## Discussion

### Summary of main findings

Our systematic review identified 15 intervention studies (11 RCTs and 4 quasi-experimental studies) where specific tasks are shared with NPHW for managing cardiovascular risk. The results of meta-analysis of RCTs demonstrate a moderate but statistically significant reduction in LDL-c and TC with task-sharing interventions. Efficacy data of task-sharing intervention on HDL-c and TG are however sparse, and the pooled estimates in their respective meta-analyses suggest no difference from usual care in terms of risk reduction. The studies used a range of strategies for task-sharing including one-to-one counseling to group education and follow-up by home visits or by telephone contacts. The overall quality of evidence available is rated as either very low or low based on GRADE criteria.

### Comparison of the effect of interventions with previous systematic reviews

Task-sharing intervention strategies have been successfully implemented in reproductive and family planning services^32^ and chronic disease management such as HIV/AIDS.^33^ The reduction in LDL-c level associated with task-sharing intervention is similar to the findings from pooled effect of multiple interventions on CVD risk reduction in LMICs.^34^ Chen et al demonstrated no effect of comprehensive lifestyle education program in patients with type II diabetes on lipid profile.^35^ Nevertheless, in our subgroup analysis, patients with type II diabetes demonstrate a reduction in LDL-c with task-sharing interventions.

### Role of NPHW involved in the intervention

Despite the involvement of high-risk patients in almost all studies, prescriptions for even the basic medications are not part of the shared interventions. One of the potential reasons may be the lack of policy on the eligibility for medicine prescription by NPHW in LMICs. On the contrary, many qualified nonphysician providers who have adequate practice credentials (eg, advanced practice nurses, nurse specialists, and advanced practice pharmacists) under the supervision of a physician can prescribe lipid-lowering medicines in Western Settings.^36^ For example, the Accreditation Council for Clinical Lipidology in the United States offers a specialization certification for Clinical Lipid Specialists. Therefore, NPHW in LMICs may benefit from additional training and accreditation in lipid management. In addition, most of the interventions included in the task-sharing strategy in LMICs are of nontechnical nature or with clear demarcation of the boundaries from the usual tasks of physicians. Expanding the scope of task-sharing and deeper involvement of NPHW in management of cardiovascular risk may be more effective in LMIC settings.

### Enablers and barriers of task-sharing interventions

Some of the enablers and barriers in implementing task-sharing intervention models as identified in our review are similar to those of Ogedegbe et al^8^ and Joshi et al.^9^ The enabling factors are the structured training of NPHW, guidelines- or algorithm-based management, and appropriateness of the intervention model in bridging the gap between hospital- and home-based care. The decision-making process of care coordinators with the help of an algorithm is a promising strategy.^25^ More active involvement of NPHW in disease management, skill building, and structured training and utilization of appropriate technologies may help to improve health outcomes. Although there are attempts to incorporate training component for the NPHW, a formal strategy to align the training with the current regulation and accreditation of trained health care worker are absent even as a policy document in most of the LMICs. Barriers include poor participant retention due to lack of adequate communication between health promoters and patients regarding the timings of health education class, difficulty in accessing care due to distance, and infrastructural limitations such as absence of a suitable space at the health care facility for group health education.

### Quality of evidence

We assessed the quality of evidence from the systematic review based on GRADE approach. For LDL-c and HDL-c analyses, we assigned the quality of evidence as “Low” because there were serious inconsistencies, indirectness, and imprecision in the pooled analyses. The quality of evidence for TC was downgraded by 2 levels due to serious levels of risk of bias and imprecision with suspected publication bias. For TG, the quality of evidence was downgraded by 3 levels because there were very serious levels of risk of bias and imprecision. Although the statistical heterogeneity was within limits, the study population, intervention, and task-sharing group were different across different trials.

### Strengths and limitations

One of the main strength of the review is the extensive search of literature in multiple databases. The inclusion of studies with different methodologies such as randomized trials and quasi-experimental studies provide more insights into factors influencing NPHW led lipid management. However, one of the limitations is possibility of missing relatively new publications. We have mitigated this by updating the search up to 30 June 2017. We also acknowledge that inclusion of studies in English only must have led to missing of articles especially in Chinese, Spanish, or other foreign languages, which do not provide abstracts in English.

We did not restrict the inclusion of studies, which targeted multiple CVD risk factors at the same time rather than managing dyslipidemia alone. Therefore, assessing effectiveness of task-sharing interventions on managing lipids alone may have underestimated the impact of global cardiovascular risk reduction. We acknowledge that this is a limitation as, in practice managing lipids is carried out along with managing overall cardiovascular risk.

### Completeness and applicability of evidence

The study population included in the selected studies is at different levels of CVD risk, and varies from obese individuals to CAD patients. Hence, we cannot confirm whether the effect of interventions can be generalized to a population level. New tasks on lifestyle modification or follow-up are shared with nurses, dietitians, and other NPHW. Lifestyle interventions evaluated mostly are general in nature with a sparse description on the structure and intensity. Detailed descriptions are not available in terms of the training given to the NPHW on lifestyle interventions. Given the low quality of studies available, well-structured task-sharing interventions that are culturally acceptable and contextually relevant need to be developed and tested in LMIC settings.

## Conclusion

Our study findings highlight scarce data on the widespread implementation and effectiveness of task-sharing strategies, specifically managing dyslipidemia in LMIC settings. Evidence from qualitative and quantitative synthesis is insufficient to state that task-sharing interventions are effective in managing dyslipidemia in LMICs. The risk of bias and small study size affected the overall evidence quality generated from RCTs, even though we demonstrate LDL-c and TC reduction with task-sharing interventions in our pooled analyses. To support task-sharing policies in LMICs, additional evidence from well-designed and adequately powered RCTs of structured intervention models are required.
