# Supplementary material for: Task-sharing interventions for cardiovascular risk reduction and lipid outcomes in low- and middle-income countries: A systematic review and meta-analysis
Source: J Clin Lipidol. 2018 May-Jun;12(3):626–42. doi: 10.1016/j.jacl.2018.02.008 (PMC5994347; doi:10.1016/j.jacl.2018.02.008)

# **Title:** **Task shifting interventions for cardiovascular risk reduction and lipid outcomes in low-middle income countries. A systematic review and meta-analysis**

**Running title: Task shifting interventions and lipids**

Authors: Anand TN*, Linju M Joseph*, Geetha A V, Joyita Chowdhury, Prabhakaran Dorairaj, Panniyammakal Jeemon^£^.

*Equal contributions

^£^Corresponding author

**Appendix 1: Search terms used for different databases.**

1.PUBMED/ Cochrane database

**CVD:** “Hyperlipidemias”[MeSH] OR “hyperlipidemias”[All Fields] OR “hyperlipidemia”[All Fields] OR “hyperlipidaemia”[All Fields] OR “hyperlipidaemias”[All Fields] OR “hyperlipemia”[All Fields] OR “hyperlipemias”[All Fields] OR “hyperlipaemia”[All Fields] OR “hyperlipaemias”[All Fields] OR “lipidemia”[All Fields] OR “lipidaemia”[All Fields] OR “high cholesterol”[All Fields] OR “hypercholesterolemia”[All Fields] OR “hypercholesterolemias”[All Fields] OR “hypercholesteremia”[All Fields] OR “hypercholesteremias”[All Fields] OR “hypercholesterolaemia”[All Fields] OR “hypercholesterolaemias”[All Fields] OR “hypercholesteraemia”[All Fields] OR “Diabetes”[All Fields] OR “diabetic”[All fields] OR "Diabetes Mellitus"[Mesh] OR “proteinuria”[Mesh] OR “proteinuria”[All Fields] OR “Albuminuria”[All Fields] OR “Hemoglobinuria”[All Fields] OR "Kidney Failure, Chronic"[Mesh] OR “chronic kidney disease”[All Fields] OR “chronic renal disease”[All Fields] OR “chronic renal insufficiency”[All Fields] OR “CKD”[All Fields] OR “end-stage renal disease”[All Fields] OR “chronic kidney failure”[All Fields] OR “chronic renal failure”[All Fields] OR “chronic kidney diseases”[All Fields] OR “chronic renal diseases”[All Fields] OR “chronic renal insufficiencies”[All Fields] OR “end-stage renal diseases”[All Fields] OR “chronic kidney failures”[All Fields] OR “chronic renal failures”[All Fields] OR “stroke”[Mesh] OR “stroke”[All Fields] OR “strokes”[All Fields] OR “brain vascular accident”[All Fields] OR “brain vascular accidents”[All Fields] OR “apoplexy”[All Fields] OR "cerebrovascular accident”[All Fields] OR "cerebrovascular accidents”[All Fields] OR “cardiomyopathies”[Mesh] OR “cardiomyopathy”[All Fields] OR “cardiomyopathies”[All Fields] OR "myocardial disease”[All Fields] OR "myocardial diseases”[All Fields] OR “myocardiopathy”[All Fields] OR “myocardiopathies”[All Fields] OR “heart neoplasms”[Mesh] OR “heart neoplasm”[All Fields] OR “heart neoplasms”[All Fields] OR "cardiac tumor”[All Fields] OR "cardiac tumors”[All Fields] OR "myocardial tumor”[All Fields] OR "myocardial tumors”[All Fields] OR "cardiac carcinoma”[All Fields] OR "cardiac carcinomas”[All Fields] OR "heart cancer”[All Fields] OR "cardiac cancers”[All Fields] OR "cardiac cancer”[All Fields] OR "heart tumor”[All Fields] OR "heart tumors”[All Fields] OR “myocardial ischemia”[Mesh] OR “myocardial ischemia”[All Fields] OR “myocardial ischemias”[All Fields] OR "ischemic heart disease”[All Fields] OR "ischemic heart diseases”[All Fields] OR “myocardial ischaemia”[All Fields] OR “myocardial ischaemias”[All Fields] OR "ischaemic heart disease”[All Fields] OR "ischaemic heart diseases”[All Fields] OR “acute coronary syndrome”[All Fields] OR “acute coronary syndromes”[All Fields] OR “coronary disease”[All Fields] OR “coronary diseases”[All Fields] OR “coronary artery disease”[All Fields] OR “coronary artery diseases”[All Fields] OR “coronary arteriosclerosis”[All Fields] OR “Coronary atherosclerosis”[All Fields] OR “coronary stenosis”[All Fields] OR “coronary stenoses”[All Fields] OR “coronary restenosis”[All Fields] OR “coronary restenoses”[All Fields] OR “coronary heart disease”[All Fields] OR “coronary heart diseases”[All Fields] OR “coronary thrombosis”[All Fields] OR “coronary thromboses” OR “coronary occlusion”[All Fields] OR “coronary occlusions”[All Fields] OR “myocardial infarction”[All Fields] OR “myocardial infarctions”[All Fields] OR “heart attack”[All Fields] OR “heart attacks”[All Fields] OR “myocardial infarct”[All Fields] OR “myocardial infarcts”[All Fields] OR “heart arrest”[Mesh] OR “heart arrest”[All Fields] OR “heart arrests”[All Fields] OR “cardiac arrest”[All Fields] OR “cardiac arrests”[All Fields] OR “asystole”[All Fields] OR “asystoles”[All Fields] OR “cardiopulmonary arrest”[All Fields] OR “cardiopulmonary arrests”[All Fields] OR

“heart failure”[Mesh] OR “heart failure”[All Fields] OR “heart failures”[All Fields] OR “cardiac failure”[All Fields] OR “cardiac failures”[All Fields] OR “myocardial failure”[All Fields] OR “myocardial failures”[All Fields] OR “heart decompensation”[All Fields] OR “hypertension”[Mesh] OR “hypertension”[All Fields] OR “hypertensions”[All Fields] OR “high blood pressure”[All Fields] OR “high blood pressures”[All Fields] OR “cardiovascular diseases”[Mesh] OR “cardiovascular disease”[All Fields] OR “cardiovascular diseases”[All Fields] OR "cardiovascular risk”[All Fields] OR "cardiovascular risks”[All Fields] OR “salt” [All Fields] OR “tobacco” [All Fields] “physical activity” [All Fields] OR “diet”[All Fields]

**Task-Shifting:** ((“Task”[All Fields] OR "tasks"[all fields]) AND ("shift"[All fields] OR "share"[All fields] OR "shifted"[all fields] OR "shifts"[all fields] OR "shifting"[all fields])) OR (shortage*[All Fields] AND ("physicians"[MeSH] OR “health personnel”[Mesh] OR "physicians"[All Fields] OR "doctors"[All Fields] OR "trained personnel"[All Fields] OR "health workforce"[All Fields] OR “health care workforce"[All Fields] OR “healthcare workforce"[All Fields] OR "health workers"[All Fields] OR "health care workers"[All Fields] OR “healthcare workers"[All Fields] OR "health care providers"[All Fields] OR “health providers"[All Fields] OR “healthcare providers"[All Fields])) OR ("nurse led"[All Fields] OR "primary health care nurse"[All Fields] OR "primary health care nurses"[All Fields] OR "primary health care nursing"[All Fields]) OR "nonphysician clinicians"[All Fields] OR "non-physician clinicians"[All Fields] OR “non physician health care workers"[All Fields] OR “nonphysician health care workers"[All Fields] OR “non physician healthcare workers"[All Fields] OR “nonphysician healthcare workers"[All Fields] OR “nonphysician health workers"[All Fields] OR “non physician health workers"[All Fields] OR ("role"[All Fields] AND ("nurse"[All Fields] OR "nurses"[all fields] OR "nursing"[all fields])) OR “community health aides”[mesh] OR “community health centers”[mesh] OR “lay health workers"[All Fields] OR “lay health care workers"[All Fields] OR “lay healthcare workers"[All Fields] OR “community health workers"[All Fields] OR “community health care workers"[All Fields] OR “community healthcare workers"[All Fields] OR “community health center”[All Fields] OR “community Health centers”[all fields] OR “community health centre”[All fields] OR “community health centres”[All Fields] OR “extended scope practitioner”[all fields] OR “extended scope practitioners”[all fields] OR “extended scope practice”[all fields] OR “enhanced role”[all fields] OR “role enhancement”[all fields] OR ((“substitution”[All Fields] OR “substituted”[All Fields] OR “substitute”[All Fields] OR “substituting”[All Fields] OR “substitutes”[All Fields] OR “delegate”[All Fields] OR “delegating”[All Fields] OR “delegates”[All Fields] OR “delegation”[All Fields] OR “delegated”[All Fields]) AND (“physicians”[mesh] OR “physician”[All Fields] OR “physicians”[All Fields] OR “doctor”[All Fields] OR “doctors”[All Fields]))

**Low-and Middle-income countries:** "developing countries"[all fields] OR "developing country"[all fields] OR "developing countries"[mesh] OR “medically underserved area”[mesh] OR “medically underserved area”[all fields] OR “medically underserved areas”[all fields] OR “low income countries”[all fields] OR “low income country”[all fields] OR “middle income countries”[all fields] OR “middle income country”[all fields] OR “global”[all fields] OR “resource poor”[all fields] OR “low resource”[all fields] OR “Africa”[Mesh] OR “Asia, Central”[Mesh] OR “Asia, Western”[Mesh] OR “Asia, Southeastern”[Mesh] OR “Indian Ocean Islands”[Mesh] OR “Central America”[Mesh] OR “South America”[Mesh] OR “Europe, Eastern”[Mesh] OR “Transcaucasia”[Mesh] OR “China”[Mesh] OR “Korea”[Mesh] OR “Mongolia”[Mesh] OR “Mexico”[Mesh] OR “Caribbean Region”[Mesh] OR “Pacific Islands”[Mesh] OR “Africa”[all fields] OR “Central Asia”[all fields] OR “western Asia”[all fields] OR “southeastern Asia”[all fields] OR “Indian Ocean Islands”[all fields] OR “Central America”[all fields] OR “South America”[all fields] OR “eastern Europe”[all fields] OR “Transcaucasia”[all fields] OR “Caribbean”[all fields] OR “Pacific Islands”[all fields] OR “Afghan”[all fields] OR “afghani”[all fields] OR “afghanistan”[all fields] OR “Bangladesh”[all fields] OR “bangladeshi”[all fields] OR “Benin”[all fields] OR “Beninese”[all fields] OR “Burkina Faso”[all fields] OR “Burkinabe”[all fields] OR “Burundi”[all fields] OR “burundian”[all fields] OR “Cambodia”[all fields] OR “cambodian”[all fields] OR “Central African Republic”[all fields] OR “central African”[all fields] OR “Chad”[all fields] OR “chadian”[all fields] OR “Comoros”[all fields] OR “comoran”[all fields] OR “Congo”[all fields] OR “congolese”[all fields] OR “Eritrea”[all fields] OR “eritrean”[all fields] OR “Ethiopia”[all fields] OR “ethiopian”[all fields] OR “Gambia”[all fields] OR “gambian”[all fields] OR “Guinea”[all fields] OR “guinean”[all fields] OR “Haiti”[all fields] OR “haitian”[all fields] OR “Kenya”[all fields] OR “Kenyan” OR “Korea”[all fields] OR “korean”[all fields] OR “Kyrgyz”[all fields] OR "kyrgyzstan"[all fields] OR “Liberia”[all fields] OR “liberian”[all fields] OR “Madagascar”[all fields] OR “malagasy”[all fields] OR “Malawi”[all fields] OR “malawian”[all fields] OR “mali”[all fields] OR “malian”[all fields] OR “mozambique”[all fields] OR “mozambican”[all fields] OR “Myanmar”[all fields] OR “myanmarese”[all fields] OR “burmese”[all fields] OR “Nepal”[all fields] OR “Nepalese”[all fields] OR “Niger”[all fields] OR “nigerian”[all fields] OR “Rwanda”[all fields] OR “rwandan”[all fields] OR “Sierra Leone”[all fields] OR “sierra leonean”[all fields] OR “Somalia”[all fields] OR “somalian”[all fields] OR “Tajikistan”[all fields] OR “tajik”[all fields] OR “tadzhik”[all fields] OR” Tanzania”[all fields] OR “tanzanian”[all fields] OR

“Togo”[all fields] OR “togolese”[all fields] OR “Uganda”[all fields] OR “ugandan”[all fields] OR “Zimbabwe”[all fields] OR “zimbabwean”[all fields] OR “Angola”[all fields] OR “angolan”[all fields] OR “Armenia”[all fields] OR “armenian”[all fields] OR “Belize”[all fields] OR “belizean”[all fields] OR “Bhutan”[all fields] OR “bhutanese”[all fields] OR “Bolivia”[all fields] OR “bolivian”[all fields] OR “Cameroon”[all fields] OR “cameroonian”[all fields] OR “Cape Verde”[all fields] OR “cape verdian”[all fields] OR “cape verdean”[all fields] OR “Côte d'Ivoire” [all fields] OR "ivory coast"[all fields] “ivorian”[all fields] OR “Djibouti”[all fields] OR “Egypt”[all fields] OR “egyptian”[all fields] OR “El Salvador”[all fields] OR “salvadoran”[all fields] OR “Fiji”[all fields] OR “fijian”[all fields] OR “Georgia”[all fields] OR “georgian”[all fields] OR “Ghana”[all fields] OR “ghanaian”[all fields] OR “Guatemala”[all fields] OR “Guatemalan”[all fields] OR “Guyana”[all fields] OR “guyanese”[all fields] OR “Honduras” OR “honduran”[all fields] OR “Indonesia”[all fields] OR “indonesian”[all fields] OR “India”[all fields] OR “indian”[all fields] OR “Iraq”[all fields] OR “iraqi”[all fields] OR “Kiribati”[all fields] OR “Kosovo”[all fields] OR “kosovar”[all fields] OR “Laos”[all fields] OR “lao”[all fields] OR “laotian”[all fields] OR “Lesotho”[all fields] OR “Marshall Islands”[all fields] OR “marshallese”[all fields] OR “Mauritania”[all fields] OR “mauritanian”[all fields] OR “Micronesia”[all fields] OR “micronesian”[all fields] OR “Moldova”[all fields] OR “moldovan”[all fields] OR “Mongolia”[all fields] OR “mongolian”[all fields] OR “Morocco”[all fields] OR “moroccan”[all fields] OR “Nicaragua”[all fields] OR “nicaraguan”[all fields] OR “Nigeria”[all fields] OR “nigerian”[all fields] OR “Pakistan”[all fields] OR “pakistani”[all fields] OR “Papua New Guinea”[all fields] OR “papua new guinean”[all fields] OR “Paraguay”[all fields] OR “paraguayan”[all fields] OR “Philippines”[all fields] OR “filipino”[all fields] OR “Samoa”[all fields] OR “samoan”[all fields] OR “Sao Tome and Principe”[all fields] OR "São Tomé and Príncipe"[all fields] OR “santomean”[all fields] OR “Senegal”[all fields] OR “senegalese”[all fields] OR “Solomon Islands”[all fields] OR “Solomon islander”[all fields] OR “Sri Lanka”[all fields] OR “sri lankan”[all fields] OR “Sudan”[all fields] OR “sudanese”[all fields] OR “Swazi”[all fields] OR “swaziland”[all fields] OR “Syria”[all fields] OR “syrian”[all fields] OR “east Timor”[all fields] OR “east timorese”[all fields] OR “Tonga”[all fields] OR “tongan”[all fields] OR “Turkmenistan”[all fields] OR “turkmen”[all fields] OR “Tuvalu”[all fields] OR “tuvaluan”[all fields] OR “Ukraine”[all fields] OR “ukrainian”[all fields] OR “Uzbekistan”[all fields] OR “uzbek”[all fields] OR “Vanuatu”[all fields] OR “Vietnam”[all fields] OR “vietnamese”[all fields] OR “West Bank”[all fields] OR “Gaza”[all fields] OR “Yemen”[all fields] OR “yemeni”[all fields] OR “yemenite”[all fields] OR “Zambia”[all fields] OR “zambian”[all fields] OR “Albania”[all fields] OR “albanian”[all fields] OR “Algeria”[all fields] OR “algerian”[all fields] OR “Antigua and Barbuda”[all fields] OR “antiguan”[all fields] OR “barbudan”[all fields] OR “Argentina”[all fields] OR “argentinian”[all fields] OR “Azerbaijan”[all fields] OR “azerbaijani”[all fields] OR “Belarus”[all fields] OR “belarusian”[all fields] OR “Bosnia”[all fields] OR “bosnian”[all fields] OR “Botswana”[all fields] OR “Brazil”[all fields] OR “brazilian”[all fields] OR “Bulgaria”[all fields] OR “bulgarian”[all fields] OR “Chile”[all fields] OR “chilean”[all fields] OR “China”[all fields] OR “Chinese”[all fields] OR “Colombia”[all fields] OR “colombian”[all fields] OR “Costa Rica”[all fields] OR “costa rican”[all fields] OR “Cuba”[all fields] OR “Cuban”[all fields] OR “Dominica”[all fields] OR “dominican”[all fields] OR “Ecuador”[all fields] OR “ecuadorean”[all fields] OR “Gabon”[all fields] OR “gabonese”[all fields] OR “Grenada”[all fields] OR “grenadian”[all fields] OR “Iran”[all fields] OR “iranian”[all fields] OR “Jamaica”[all fields] OR “jamaican”[all fields] OR “Jordan”[all fields] OR “jordanian”[all fields] OR “Kazakhstan”[all fields] OR “kazakhstani”[all fields] OR “Latvia”[all fields] OR “latvian”[all fields] OR “Lebanon”[all fields] OR “lebanese”[all fields] OR “Libya”[all fields] OR “libyan”[all fields] OR “Lithuania”[all fields] OR “lithuanian”[all fields] OR “Macedonia”[all fields] OR “macedonian”[all fields] OR “Malaysia”[all fields] OR “malaysian”[all fields] OR “Maldives”[all fields] OR “maldivian”[all fields] OR “mauritius”[all fields] OR “mauritian”[all fields] OR “Mexico”[all fields] OR “mexican”[all fields] OR “Montenegro”[all fields] OR “montenegrin”[all fields] OR “Namibia”[all fields] OR “namibian”[all fields] OR “Palau”[all fields] OR “palauan”[all fields] OR “Panama”[all fields] OR “panamanian”[all fields] OR “Peru”[all fields] OR “peruvian”[all fields] OR “Romania”[all fields] OR “romanian”[all fields] OR “Russia”[all fields] OR “russian”[all fields] OR “Serbia”[all fields] OR “serbian”[all fields] OR “Seychelles”[all fields] OR “seychellois”[all fields] OR “South Africa”[all fields] OR “south african”[all fields] OR “Saint Kitts”[all fields] OR “saint Lucia”[all fields] OR “Saint Vincent”[all fields] OR “Suriname”[all fields] OR “surinamer”[all fields] OR “thailand”[all fields] OR “Thai”[all fields] OR “Tunisia”[all fields] OR “tunisian”[all fields] OR “Turkey”[all fields] OR “turkish”[all fields] OR “Uruguay”[all fields] OR “uruguayan”[all fields] OR “Venezuala”[all fields] OR “venezualan”[all fields] OR “Sub Saharan Africa”[all fields]

**2. PsycInfo Search Terms**

|  | **Searches** | **Results** |
| --- | --- | --- |
| 1 | hyperlipidemia*.ab. or hyperlipidemia*.ti. | 726 |
| 2 | hyperlipidaemia*.ab. or hyperlipidaemia*.ti. | 75 |
| 3 | hyperlipemia*.ab. or hyperlipemia*.ti. | 15 |
| 4 | hyperlip?emia*.ab. or hyperlip?emia*.ti. | 18 |
| 5 | lipidemia*.ab. or lipidemia*.ti. | 9 |
| 6 | lipid?emia*.ab. or lipid?emia*.ti. | 10 |
| 7 | high cholesterol.ab. or high cholesterol.ti. | 356 |
| 8 | hypercholesterolemia*.ab. or hypercholesterolemia*.ti. | 537 |
| 9 | hypercholesterol?emia*.ab. or hypercholesterol?emia*.ti. | 631 |
| 10 | hypercholester?emia*.ab. or hypercholester?emia*.ti. | 8 |
| 11 | diabet*.ab. or diabet*.ti. | 22656 |
| 12 | proteinuria.ab. or proteinuria.ti. | 121 |
| 13 | albuminuria.ab. or albuminuria.ti. | 56 |
| 14 | hemoglobinuria.ab. or hemoglobinuria.ti. | 6 |
| 15 | chronic kidney*.ab. or chronic kidney*.ti. | 477 |
| 16 | chronic renal*.ab. or chronic renal*.ti. | 418 |
| 17 | CKD.ab. or CKD.ti. | 215 |
| 18 | end-stage renal*.ab. or end-stage renal*.ti. | 795 |
| 19 | stroke*.ab. or stroke*.ti. | 25019 |
| 20 | brain vascular accident*.ab. or brain vascular accident*.ti. | 0 |
| 21 | apoplexy.ab. or apoplexy.ti. | 119 |
| 22 | cerebrovascular accident*.ab. or cerebrovascular accident*.ti. | 699 |
| 23 | cardiomyopath*.ab. or cardiomyopath*.ti. | 535 |
| 24 | myocardial disease*.ab. or myocardial disease*.ti. | 9 |
| 25 | myocardiopath*.ab. or myocardiopath*.ti. | 8 |
| 26 | myocardial isch?emia*.ab. or myocardial isch?emia*.ti. | 248 |
| 27 | myocardial ischemia*.ab. or myocardial ischemia*.ti. | 229 |
| 28 | isch?emic heart disease*.ab. or isch?emic heart disease*.ti. | 835 |
| 29 | acute coronary syndrome*.ab. or acute coronary syndrome*.ti. | 431 |
| 30 | coronary disease*.ab. or coronary disease*.ti. | 368 |
| 31 | coronary artery disease*.ab. or coronary artery disease*.ti. | 1578 |
| 32 | coronary arteriosclerosis.ab. or coronary arteriosclerosis.ti. | 7 |
| 33 | coronary atherosclerosis.ab. or coronary atherosclerosis.ti. | 96 |
| 34 | coronary stenos?s.ab. or coronary stenos?s.ti. | 20 |
| 35 | coronary restenos?s.ab. or coronary restenos?s.ti. | 1 |
| 36 | coronary heart disease*.ab. or coronary heart disease*.ti. | 3569 |
| 37 | coronary thrombos?s.ab. or coronary thrombos?s.ti. | 21 |
| 38 | coronary occlusion*.ab. or coronary occlusion*.ti. | 42 |
| 39 | myocardial infarction*.ab. or myocardial infarction*.ti. | 3543 |
| 40 | heart attack*.ab. or heart attack*.ti. | 844 |
| 41 | myocardial infarct*.ab. or myocardial infarct*.ti. | 3615 |
| 42 | heart arrest*.ab. or heart arrest*.ti. | 7 |
| 43 | cardiac arrest*.ab. or cardiac arrest*.ti. | 625 |
| 44 | asystole*.ab. or asystole*.ti. | 85 |
| 45 | cardiopulmonary arrest*.ab. or cardiopulmonary arrest*.ti. | 62 |
| 46 | heart failure*.ab. or heart failure*.ti. | 2662 |
| 47 | cardiac failure*.ab. or cardiac failure*.ti. | 109 |
| 48 | myocardial failure*.ab. or myocardial failure*.ti. | 1 |
| 49 | hypertension*.ab. or hypertension*.ti. | 11808 |
| 50 | high blood pressure*.ab. or high blood pressure*.ti. | 1141 |
| 51 | cardiovascular*.ab. or cardiovascular*.ti. | 21768 |
| 52 | cardio?vascular*.ab. or cardio?vascular*.ti. | 21769 |
| 53 | 1 or 2 or 3 or 4 or 5 or 6 or 7 or 8 or 9 or 10 or 11 or 12 or 13 or 14 or 15 or 16 or 17 or 18 or 19 or 20 or 21 or 22 or 23 or 24 or 25 or 26 or 27 or 28 or 29 or 30 or 31 or 32 or 33 or 34 or 35 or 36 or 37 or 38 or 39 or 40 or 41 or 42 or 43 or 44 or 45 or 46 or 47 or 48 or 49 or 50 or 51 or 52 | 81290 |
| 54 | task shifting.ab. or task shifting.ti. | 134 |
| 55 | task sharing.ab. or task sharing.ti. | 70 |
| 56 | task-shift*.ab. or task-shift*.ti. | 187 |
| 57 | task-sharing.ab. or task-sharing.ti. | 70 |
| 58 | task shifting.mp. | 148 |
| 59 | task sharing.mp. | 85 |
| 60 | (task and shift*).ti. | 241 |
| 61 | (task adj shift*).ab. | 180 |
| 62 | (task and shar*).ti. | 129 |
| 63 | (task adj sharing).ab. | 65 |
| 64 | ((role* or substitut* or shortage* or delegat*) adj (physician* or health personnel* or doctor* or nurs* or trained personnel* or health* workforce* or nonphysician* or non-physician* or "health* worker*" or "community health aid*" or "extended scope practitioner*")).ab. or ((role* or substitut* or shortage* or delegat*) adj (physician* or health personnel* or doctor* or nurs* or trained personnel* or health* workforce* or nonphysician* or non-physician* or "health* worker*" or "community health aid*" or "extended scope practitioner*")).ti. | 151 |
| 65 | ((role* or substitut* or shortage* or delegat*) and (physician* or health personnel* or doctor* or nurs* or trained personnel* or health* workforce* or nonphysician* or non-physician* or "health* worker*" or "community health aid*" or "extended scope practitioner*")).ab. or ((role* or substitut* or shortage* or delegat*) and (physician* or health personnel* or doctor* or nurs* or trained personnel* or health* workforce* or nonphysician* or non-physician* or "health* worker*" or "community health aid*" or "extended scope practitioner*")).ti. | 23899 |
| 66 | 54 or 55 or 56 or 57 or 58 or 59 or 60 or 61 or 62 or 63 or 64 or 65 | 24444 |
| 67 | developing countr*.af. | 27081 |
| 68 | developing countries.sh. | 4468 |
| 69 | medically underserved area.af. | 20 |
| 70 | medically underserved area.sh. | 0 |
| 71 | low income countr*.af. | 2720 |
| 72 | low income countries.sh. | 0 |
| 73 | middle income countr*.af. | 4732 |
| 74 | middle income countries.sh. | 0 |
| 75 | global.af. | 262056 |
| 76 | resource poor.af. | 1626 |
| 77 | low resource.af. | 827 |
| 78 | (Africa* or Central Asia* or Western Asia* or Southeastern Asia* or Indian Ocean Island* or Central America* or South America* or Eastern Europe* or Transcaucasia* or Chin* or Korea* or Mongolia* or Mexic* or Carribean region* or Pacific Island*).af. | 467610 |
| 79 | (Afghan* or Bangladesh* or Benin* or Burkina Faso or Burkinabe or Burundi* or Cambodia* or Central African* Republic or Chad* or Comor* or Congo* or Eritrea* or Ethiopia* or Gambia* or Guinea* or Haiti* or Kenya* or Kyrgyz* or Liberia*).af. | 210072 |
| 80 | (Madagas* or Malawi* or Mali* or Mozambi* or Myanmar* or Burmese or Nepal* or Niger* or Rwanda* or Sierra Leone* or Somalia* or Tajik* or Tadzhik or Tanzania* or Togo* or Uganda* or Zimbabwe* or Angola* or Armenia* or Belize* or Bhutan*).af. | 95283 |
| 81 | (Bolivia* or Cameroon* or Cape Verd* or Cote d'Ivoire or Ivory cost or Ivorian or Djibouti or Egypt* or El Salvador* or Salvadorian or Fiji* or Georgia* or Ghana* or Guatemala* or Guyan* or Hondura* or Indonesia* or India* or Iraq* or Kiribati or Kosov* or Lao* or Lesotho).af. | 254248 |
| 82 | (Marshall Islands or Marshallese or Mauritania* or Micronesia* or Moldova* or Mongolia* or Morocc* or Nicaragua* or Nigeria* or Pakistan* or Papua New Guinea* or Paraguay* or Philippines or Filipino or Samoa* or (Sao Tome and Principe) or santomean or Senegal* or Solomon Island* or Sri Lanka* or Sudan* or Swazi* or Syria*).af. | 63950 |
| 83 | (Libya* or Lithuania* or Macedonia* or Malaysia* or Maldiv* or Mauriti* or Montenegr* or Namibia* or Palau* or Panama* or Peru* or Romania*).af. | 50753 |
| 84 | (East Timor* or Tonga* or Turkmen* or Tuvalu* or Ukrain* or Uzbek* or Vanuatu or Vietnam* or West Bank or Gaza or Yemen* or Zambia* or Albania* or Algeria* or (Antigua* and Barbuda*) or Azerbaijan* or Belarus* or Bosnia* or Botswana* or Brazil* or Bulgaria* or Colombia*).af. | 128658 |
| 85 | (Costa Rica* or Cuba* or Dominica* or Ecuador* or Gabon* or Grenada* or Iran* or Jamaica* or Jordan* or Kazakhstan* or Leban*).af. | 81635 |
| 86 | (Russia* or Serbia* or Seychell* or South Africa* or Saint Kitts or Saint Lucia or Saint Vincent or Suriname* or Thai* or Tunisia* or Turk* or Uruguay*).af. | 188797 |
| 87 | 67 or 68 or 69 or 70 or 71 or 72 or 73 or 74 or 75 or 76 or 77 or 78 or 79 or 80 or 81 or 82 or 83 or 84 or 85 or 86 | 1137564 |
| 88 | 53 and 66 and 87 | 398 |
| **89** | **from 88 keep 1-398** | **398** |

**3. Search terms for CINAHL**

**CVD:** (MH “cardiovascular diseases+”) OR (TX cardiovascular N5 disease#) OR (MH “cardiovascular

risk factors+”) OR (TX cardiovascular N5 risk#) OR (MH “Hyperlipidemia+”) OR (TX hyperlipid#emia#)

OR (TX hyperlip#emia#) OR (TX lipid#emia#) OR (TX "high cholesterol") OR (TX

hypercholesterol#emia#) OR (TX hypercholester#emia#) OR (TX diabetes) OR (TX diabetic) OR (MH

“Diabetes Mellitus+”) OR (MH “proteinuria+”) OR (TX proteinuria#) OR (TX albuminuria#) OR (TX

hemoglobinuria#) OR (MH "Kidney Failure, Chronic") OR (TX “chronic kidney disease#”) OR (TX

“chronic renal disease#”) OR (TX “chronic renal insufficienc*”) OR (TX "CKD") OR (TX “end-stage

renal disease#”) OR (TX “chronic kidney failure#”) OR (TX “chronic renal failure#”) OR (TX stroke#) OR

(TX "brain vascular accident#") OR (TX apoplexy*) OR (TX "cerebrovascular accident#") OR (TX

cardiomyopath*) OR (TX “myocardial disease#”) OR (TX myocardiopath*) OR (TX "heart neoplasm#")

OR (TX "cardiac tumor#") OR (TX "myocardial tumor#") OR (TX "cardiac carcinoma#") OR (TX

"heart cancer#") OR (TX "cardiac cancer#") OR (TX "heart tumor#") OR (TX "myocardial isch#emia#")

OR (TX "isch#emic heart disease#") OR (TX “acute coronary syndrome#”) OR (TX “coronary disease#”

) OR (TX “coronary artery disease#”) OR (TX “coronary arterioscleros*”) OR (TX “coronary

atheroscleros*”) OR (TX “coronary stenos*”) OR (TX “coronary restenos*”) OR (TX “coronary heart

disease*”) OR (TX “coronary thrombos*”) OR (TX “coronary occlusion#”) OR (TX “myocardial

infarct*”) OR (TX “heart attack#") OR (TX "heart arrest#") OR (TX "cardiac arrest#") OR (TX

asystole#) OR (TX "cardiopulmonary arrest#") OR (TX "heart failure#") OR (TX "cardiac failure#") OR

(TX "myocardial failure#") OR (TX "heart decompensation#") OR (TX hypertension#) OR (TX "high

blood pressure#")

**Task-Shifting:** (MH “Personnel Shortage+”) OR (TX shortage# N5 doctor#) OR (TX shortage# N5

physician#) OR (TX shortage# N5 “trained personnel”) OR (TXshortage# N5 “health* * workforce”) OR

(TX shortage# N5 “health* * worker#”) OR (TX shortage# N5 “health* * provider#”) OR (TX task# N5

shift*) OR (TX "nurse led") OR (TX "non*physician clinicians") OR (TX “non*physician health* *

worker#”) OR (TX "primary health* * nurs*") OR (TX role N5 nurs*) OR (MH “community health

workers+”) OR (MH “community health centers+”) OR (TX "lay health* * worker#") OR (TX community

N2 “health* * aide#”) OR (TX community N2 “health* * worker#”) OR (TX community N2 “health* *

cent*") OR (TX "extended scope practi*") OR (TX role N3 enhance*) OR (TX substitut* N10

physician#) OR (TX substitut* N10 doctor#) OR (TX substitute* N10 nurse#) OR (TX delegat* N10

physician#) OR (TX delegat * N10 doctor#) OR (TX delegat * N10 nurse#)

**Low-and Middle-income countries:** (MH “developing countries+”) OR (MH “medically underserved

area+”) OR (TX "developing countr*") OR (TX "medically underserved area#") OR (TX "low income

countr *") OR (TX "middle income countr*") OR (MH “Africa+”) OR (TX Africa#) OR (TX Caribbean)

OR (MH “west indies+”) OR (TX “central America#”) OR (MH “Central America+”) OR (TX “south

America#”) OR (MH “south America+”) OR (TX global) OR (TX “low resource”) OR (TX “resource

poor”) OR (TX “central asia#”) OR (MH “asia, central+”) OR (TX “southeastern asia#”) OR (MH "asia,

southeastern+”) OR (TX “western asia#”) OR (MH “asia, western+”) OR (TX “Indian ocean islands”) OR

(MH “Indian ocean islands+”) OR (TX “eastern Europe*”) OR (MH “europe, eastern”) OR (TX

Transcaucasia#) OR (TX “pacific islands” )OR (MH “pacific islands+”) OR (TX Afghan*) OR (TX

Bangladesh#) OR (TX Benin*) OR (TX “Burkina Faso” ) OR (TX burkinabe) OR (TX Burundi*) OR

(TX Cambodia#) OR (TX “Central African” ) OR (TX Chad*) OR (TX Comor*) OR (TX Congo*) OR

(TX Eritrea#) OR (TX Ethiopia#) OR (TX Gambia#) OR (TX Guinea#) OR (TX Haiti*) OR (TX

Kenya#) OR (TX Korea#) OR (TX Kyrgyz*) OR (TX Liberia#) OR (TX Madagascar) OR (TX

malagasy) OR (TX Malawi*) OR (TX mali*) OR (TX mozambi*) OR (TX Myanmar*) OR (TX

Nepal*) OR (TX Niger*) OR (TX Rwanda#) OR (TX “Sierra Leon*”) OR (TX Somalia#) OR (TX

Tajik*) OR (TX tadzhik) OR (TX Tanzania#) OR (TX Togo*) OR (TX Uganda#) OR (TX Zimbabwe*

) OR (TX Angola#) OR (TX Armenia#) OR (TX Belize*) OR (TX Bhutan*) OR (TX Bolivia#) OR

(TX Cameroon*) OR (TX Cape Verd*) OR (TX Congo*) OR (TX “Côte d'Ivoire” ) OR (TX "ivory

coast") OR (TX ivorian) OR (TX Djibouti) OR (TX Egypt#) OR (TX “El Salvador” ) OR (TX

“Salvadoran” ) OR (TX Fiji*) OR (TX Georgia#) OR (TX Ghana*) OR (TX Guatemala#) OR (TX

Guyan*) OR (TX Hondura#) OR (TX Indonesia#) OR (TX India#) OR (TX Iraq#) OR (TX Kiribati)

OR (TX Kosov*) OR (TX Lao*) OR (TX Lesotho) OR (TX (“Marshall Islands”) ) OR (TX

marshallese) OR (TX Mauritania#) OR (TX Micronesia#) OR (TX Moldova#) OR (TX Mongolia#) OR

(TX Morocc*) OR (TX Nicaragua#) OR (TX Nigeria#) OR (TX Pakistan#) OR (TX “Papua New Guinea*”) OR (TX Paraguay*) OR (TX Philippines) OR (TX Filipino) OR (TX Samoa#) OR (TX “Sao

Tome*”) OR (TX Senegal*) OR (TX “Solomon Island*”) OR (TX “sri lanka#”) OR (TX Sudan*) OR

(TX Swazi*) OR (TX Syria#) OR (TX Timor*) OR (TX Tonga#) OR (TX Turkmen*) OR (TX

Tuvalu*) OR (TX Ukrain*) OR (TX Uzbek*) OR (TX Vanuat*) OR (TX Vietnam*) OR (TX (“West

Bank”) ) OR (TX Gaza) OR (TX Yemen*) OR (TX Zambia#) OR (TX Albania#) OR (TX Algeria#) OR

(TX “Antigua and Barbuda” ) OR (TX antiguan) OR (TX barbudan) OR (TX Argentin*) OR (TX

Azerbaijani*) OR (TX Belarus*) OR (TX Bosnia#) OR (TX Botswana) OR (TX Brazil*) OR (TX

Bulgaria#) OR (TX Chile*) OR (TX China) OR (TX Chinese) OR (MH "China+") OR (TX Colombia#)

OR (TX “Costa Rica#”) OR (TX Cuba#) OR (TX Dominica#) OR (TX Ecuador*) OR (TX Gabon*) OR

(TX Grenad*) OR (TX Iran*) OR (TX Jamaica#) OR (TX Jordan*) OR (TX Kazakhstan#) OR (TX

Latvia#) OR (TX Leban*) OR (TX Libya#) OR (TX Lithuania#) OR (TX Macedonia#) OR (TX

Malaysia#) OR (TX Maldiv*) OR (TX mauriti*) OR (TX Mexic*) OR (TX Montenegr*) OR (TX

Namibia#) OR (TX Palau*) OR (TX Panama*) OR (TX Peru*) OR (TX Romania#) OR (TX Russia#)

OR (TX Serbia#) OR (TX Seychell*) OR (TX “South Africa#”) OR (TX “Saint Kitts” ) OR (TX “Saint

Lucia” ) OR (TX “Saint Vincent” ) OR (TX Suriname#) OR (TX Thai*) OR (TX Tunisia#) OR (TX

Turk*) OR (TX Uruguay*) OR (TX Venezuala#)

**4. Search terms for EMBASE**

**CVD:** exp hyperlipidemia/ or hyperlipid?emia$1.mp. or hyperlip?emia$1.mp. or lipid?emia$1.mp. or high

cholesterol.mp. or hypercholesterol?emia$1.mp. or hypercholester?emia$1.mp. or exp Diabetes mellitus/

or diabetes.mp. OR exp diabetic angiopathy/ or diabetic.mp. or exp proteinuria/ or proteinuria$1.mp. or

albuminuria$1.mp. or hemoglobinuria$1.mp. or exp chronic kidney disease/ or chronic kidney

disease$1.mp. or chronic renal disease$1.mp. or chronic renal insufficienc$.mp. OR CKD.mp. OR endstage

renal disease$1.mp. or chronic kidney failure$1.mp. or chronic renal failure$1.mp. or exp stroke/ or

stroke$1.mp. or brain vascular accident$1.mp. or apoplexy.mp. or cerebrovascular accident$1.mp. or exp

myocardial disease/ or cardiomyopath$.mp. or myocardial disease$1.mp. or myocardiopath$.mp. or heart

muscle isch?emia$1.mp. or myocardial isch?emia$1.mp. or isch?emic heart disease$1.mp. or acute

coronary syndrome$1.mp. or coronary disease$1.mp. or coronary artery disease$1.mp. or coronary

arterioscleros$.mp. or coronary atheroscleros$.mp. or coronary stenos$.mp. or coronary restenos$.mp. or

coronary heart disease$1.mp. or coronary thrombos$.mp. or coronary occlusion$1.mp. or myocardial

infarct$.mp. or heart attack$1.mp. or exp heart tumor/ or heart neoplasm$1.mp. or cardiac tumor$1.mp. or

myocardial tumor$1.mp. or cardiac carcinoma$1.mp. or heart cancer$1.mp. or cardiac cancer$1.mp. or

heart tumor$1.mp. or exp heart failure/ or heart arrest$1.mp. or cardiac arrest$1.mp. or asystole$1.mp. or

cardiopulmonary arrest$1.mp. or heart failure$1.mp. or cardiac failure$1.mp. or myocardial failure$1.mp.

or heart decompensation$1.mp. or exp hypertension/ or hypertension$1.mp. or high blood pressure$1.mp.

or exp cardiovascular disease/ or exp cardiovascular risk/ or (cardiovascular ADJ5 disease$1).mp. or

(cardiovascular ADJ5 risk$1).mp.

**Task-Shifting:** exp personnel shortage/ or (shortage$1 ADJ5 doctor$1).mp. or (shortage$1 ADJ5

physician$1).mp. or (shortage$1 ADJ5 trained ADJ5 personnel).mp. or (shortage$1 ADJ5 health ADJ5

workforce).mp. or (shortage$1 ADJ5 health ADJ5 worker$1).mp. or (shortage$1 ADJ5 health ADJ5

provider$1).mp. or (task$1 ADJ5 shift$).mp. or nurse led.mp. or non$1physician clinician$1.mp. or

non$1physician health$ worker$1.mp. or primary health care nurs$.mp. or (role ADJ5 nurs$).mp. or exp

community health nursing/ or exp health auxiliary/ or community health$ worker$1.mp. or community

health cent$.mp. or lay health$ worker$1.mp. or community health$ aide$1.mp. or (community ADJ2

health ADJ5 worker$1).mp. or extended scope practi$.mp. or (role ADJ3 enhance$).mp. or (substitute$

ADJ10 physician$1).mp. or (substitute$ ADJ10 doctor$1).mp. or (substitute$ ADJ10 nurse$1).mp. or

(delegat$ ADJ10 physician$1).mp. or (delegat$ ADJ10 doctor$1).mp. or (delegat$ ADJ10 nurse$1).mp.

**Low-and Middle-income countries:** exp developing country/ or exp medically underserved/ or developing

countr$.mp. or medically underserved area$1.mp. or low income countr$.mp. or middle income country.mp. or low resource.mp. or resource poor.mp. or global.mp. or exp Africa/ or exp "South and

Central America"/or exp asia/ or exp Caribbean islands/ or exp pacific islands/ or exp eastern Europe/ or

exp Indian Ocean/or south america$1.mp. or Africa$1.mp. or Caribbean.mp. or central America$1.mp. or

south America$1.mp. or eastern Europe$1.mp. or pacific island$.mp. or Indian ocean island$.mp. or

asia.mp. or Afghan$.mp. or Bangladesh$1.mp. or Benin$.mp. or Burkina Faso.mp. or Burkinabe.mp. or

Burundi$.mp. or Cambodia$1.mp. or Central African.mp. or Chad$.mp. or Comor$.mp. or Congo$.mp. or

Eritrea$1.mp. or Ethiopia$1.mp. or Gambia$1.mp. or Guinea$1.mp. or Haiti$.mp. or Kenya$1.mp. or

Korea$1.mp. or exp North Korea/ or Kyrgyz$.mp. or Liberia$1.mp. or Madagascar.mp. or Malagasy.mp. or

Malawi$.mp. or mali$.mp. or mozambi$.mp. or Myanmar$.mp. or Nepal$.mp. or Niger$.mp. or

Rwanda$1.mp. or Sierra Leone$.mp. or Somalia$1.mp. or Tajik$.mp. or Tanzania$1.mp. or Togo$.mp. or

Uganda$1.mp. or Zimbabwe$.mp. or Angola$1.mp. or Armenia$1.mp. or Beliz$.mp. or Bhutan$.mp. or

Bolivia$1.mp. or Cameroon$.mp. or Cape Verde$.mp. or Congo$.mp. or "Côte d'Ivoire".mp. or Ivory

Coast.mp. or Ivorian.mp. or Djibouti.mp. or Egypt$.mp. or El Salvador.mp. or Salvadoran.mp. or

Fiji$.mp. or Georgia$1.mp. or Ghana$.mp. or Guatemala$1.mp. or Guyan$.mp. or Hondura$.mp. or

Indonesia$1.mp. or India$1.mp. or Iraq$1.mp. or Kiribati.mp. or Kosov$.mp. or Lao$.mp. or Lesotho.mp.

or Marshall Islands.mp. or Marshallese.mp. or Mauritania$1.mp. or Micronesia$1.mp. or Moldov$.mp. or

Mongolia$1.mp. or Morocc$.mp. or Nicaragua$1.mp. or Nigeria$1.mp. or Pakistan$1.mp. or Papua New

Guinea$1.mp. or Paraguay$.mp. or Philippines.mp. or Filipino.mp. or Samoa$1.mp. or sao tome$.mp. or

Senegal$.mp. or Solomon Island$.mp. or sri lanka$1.mp. or Sudan$.mp. or Swazi$.mp. or Syria$1.mp. or

Timor$.mp. or Tonga$1.mp. or Turkmen$.mp. or Tuvalu$.mp. or Ukrain$.mp. or Uzbek$.mp. or

Vanuat$1.mp. or Vietnam$.mp. or West Bank.mp. or Gaza.mp. or Yemen$.mp. or Zambia$1.mp. or

Albania$1.mp. or Algeria$1.mp. or "Antigua and Barbuda".mp. or antiguan.mp. or barbudan.mp. or

Argentin$.mp. or Azerbaijan$1.mp. or Belarus$.mp. or Bosnia$1.mp. or Botswana.mp. or Brazil$.mp. or

Bulgaria$1.mp. or Chile$.mp. or China.mp. or Chinese.mp. or Colombia$1.mp. or Costa Rica$1.mp. or

Cuba$1.mp. or Dominica$1.mp. or Ecuador$.mp. or Gabon$.mp. or Grenad$.mp. or Iran$.mp. or

Jamaica$1.mp. or Jordan$.mp. or Kazakhstan$1.mp. or Latvia$1.mp. or Leban$.mp. or Libya$1.mp. or

Lithuania$1.mp. or Macedonia$1.mp. or Malaysia$1.mp. or Maldiv$.mp. or mauriti$.mp. or Mexic$.mp. or

Montenegr$.mp. or Namibia$1.mp. or Palau$.mp. or Panama$.mp. or Peru$.mp. or Romania$1.mp. or

Russia$1.mp. or Serbia$1.mp. or Seychell$.mp. or South Africa$1.mp. or Saint Kitts.mp. or Saint

Lucia.mp. or Saint Vincent.mp. or Suriname$1.mp. or Thai$.mp. or Tunisia$1.mp. or Turk$.mp. or Uruguay$.mp. or Venezuala$1.mp.

**Appendix 2: Research Protocol**

**A systematic review and meta-analysis of task shifting or task sharing interventions for managing dyslipidaemia in LMICs.**

**Background and Rationale**

Low Middle Income Countries (LMIC) now battle double burden of disease with an increase in non-communicable diseases(NCD) to an already existing infectious disease burden.^1^About 28 million NCD deaths occur in LMICs and cardiovascular diseases (CVD) are major contributor towards the disease burden in LMICs.^2^In resource, constrained settings with challenges of skilled work force it would be sensible in utilising the existing health care workers for screening and management of CVDs which is likely lead to more physician time available for the urgent cases.

Major risk factors for CVD include hypertension (high blood pressure), overweight (body mass index ranges from 25.0 – 29.9kg/m^2^) or obesity (body mass index ≥ 30 kg/m^2^), dyslipidemia and use of tobacco. These risk factors affect CVD-related morbidity and mortality, which can be improved by early recognition of those who are at risk, or have undiagnosed disease.^3^

Previous review by Ogedegbe et al which included RCTs informed that there is some evidence of the effectiveness of task-shifting strategies for hypertension and diabetes management using nurses in LMICs.^4^ Review by Joshi et al included task shifting interventions pertaining to non-communicable diseases.^5^ Dyslipidemia is one of the contributing factor to CVD which is often overlooked and hence it is necessary to understand how task shifting is done in managing dyslipidemia.

Similarly, previous reviews on task shifting and HIV has shown what works and this focused review will enable tailoring the interventions for CVDs. ^6^Another fact to be considered is that the healthcare work force in LMICs are strained with poor infrastructure and are usually over worked. Hence when considering to utilize their services for more interventions it should be based on priority and those interventions which requires minimal training or additional support would be better to be task shifted.^7^

The focus of current review is to identify and understand the various task shifting or task sharing interventions used in risk reduction and management of dyslipidaemia which can be further utilized to inform policy making and guide further research.

**Research questions**

The aim of this paper is to conduct a systematic review and meta-analysis to identify interventions used to task-sharing/task shifting care for people with dyslipidaemia in low- and middle-income countries (LMICs). Specific research questions are

1. What are the interventions that can be task shifted or shared?
2. Does task shifting improve dyslipidaemia outcomes?

**Methods**

**Study design**

The systematic review will be done as per the Preferred Reporting Items for Systematic Reviews and Meta-Analyses (PRISMA) guidelines.8

**Definitions**

**Task shifting**

For this review, we go by the following definition, task-shifting as the delegation of tasks to an existing or new cadre of health workers with either less training or disease-/skill-specific training. It involves shifting delivery of the task from professionals to health workers with fewer qualifications or creating a new workforce with specific training for a specific task.^9^ Also, expanding the scope of practice of an existing healthcare cadre to accept additional task or functions.

**Classification of countries**

Countries were classified into upper middle income, middle income and lower middle income using World Bank Lending Groups.^10^

**Criteria for considering studies for this review**

**Types of studies**

Specifically, quantitative studies (randomised controlled trials, quasi-experimental studies, before and after studies)

**Condition or domain being studied**

We will be studying task shifting or task sharing interventions for people at risk and with dyslipidaemia.

**Types of participants**

This review will include all adults above 18 years living in a low/low-middle income country with known dyslipidaemia or with potential risk factors such as overweight, sedentary lifestyle or with potential CVD risk factors.

**Types of interventions**

This review will include the full range of task-sharing interventions for people with CVD. Any intervention delivered by health care workers intended to screen, risk reduction and management of CVD. Studies where tasks performed by physician or nurse usually, is shifted to another health care cadre. This review will include interventions if the description was adequate for us to establish that it was a task shifted intervention.

Types of healthcare providers

Any healthcare worker (paid or voluntary) including community health workers, village health workers, nutrition workers and home visitors. Nurses, pharmacists, trained nurse aides, medical assistants, physician assistants, other paramedical workers, and trainee health professionals who were trained in some way in the context of the intervention.

Examples of studies of specific components of task-shared CVD health care that may be included in this review include:

1. At community level, community-based screening and referral, outreach to promote ongoing engagement with facility-based care, rehabilitation, and
2. At facility level, assessment, treatment, health education, adherence support and follow-up, rehabilitation goal-setting.

Any efforts or activities promoting self-care such as patient telephonic follow up may be included as previously there was no follow up and now to ensure better adherence with medication or compliance with therapy it is included.

**Exclusions**

Studies which measured only patient’s knowledge, attitudes, or intentions. Such studies considering knowledge change pre-and post without measuring any of the relevant clinical outcomes will be excluded.

Interventions which involve peer groups only were excluded as they will be more likely to informal support and will not receive any formal training for the intervention. Task-sharing activities that are exclusive to traditional healers and those that promote self-care management or informal care giver health education alone will not be included in this review.

**Types of outcome measures**

The studies will be included if they have measured and reported any of the following outcomes **-**control of lipid levels- Change in total cholesterol, LDLc, HDLc and/ triglycerides.

**Search strategy**

Search strategy will be developed following the key words identified in the review by Ogedegbe et al. The search strategy will be developed for PubMed and modified appropriately for Cochrane Library, CINAHL, EMBASE and PsycINFO with the help of a librarian. No limits on language or publication year will be applied during search.

Data collection

Two investigators (ATN and LJ) will review titles and abstract of articles identified by the search strategy. Those that appear to be studies of task shifting interventions for dyslipidemia prevention or management in a LMIC will be selected for full-text review. Two investigators (ATN and LJ) will further review full text articles independently. A third investigator (PJ) will serve as tiebreaker, independently reviewing articles to resolve disagreement between the other two investigators.

**Data management and statistical analysis**

Data extraction will be conducted by two investigators (ATN and LJ). First studies will be grouped in terms of countries of focus, the cadres discussed, the disease focus and interventions in different levels of dyslipidemia prevention and management.

Following data extraction, task shifting interventions will be summarized based on their study design. Observational study results will be synthesized narratively. Quality of individual studies will be appraised using NHLBI scale for before and after studies and cross-sectional studies. Cochrane Risk of Bias will be used for assessing quality of RCTs. Potential RCTs will be grouped to perform meta-analysis and to detect heterogeneity, we will use Q statistics and I^2^ values. Appropriate sub-group analyses will be a conducted to estimate the effect of intervention on different subgroups.

We propose to assess the quality of the evidence for each outcome across studies included in the meta-analyses following GRADE approach.

**References**

1. Tsolekile LP, Gessel SA, Puoane T. Healthcare Professional Shortage and Task-Shifting to Prevent Cardiovascular Disease: Implications for Low and Middle-Income Countries. Curr Cardiol Rep (2015) 17: 115 DOI 10.1007/s11886-015-0672-y
2. Organization WH. Cardiovascular diseases (CVDs). Updated2015.
3. Abrahams-Gessel S, Denman CA, Montano CM, et al. The Training and Field Work Experiences of Community Health Workers conducting non-invasive, population-based screening for Cardiovascular Disease in Four Communities in Low and Middle-Income Settings. *Global heart*. 2015;10(1):45-54. doi:10.1016/ j. gheart.2014.12.008
4. Ogedegbe G, Gyamfi J, Plange-Rhule J, et al. Task shifting interventions for cardiovascular risk reduction in low-income and middle income countries: a systematic review of randomised controlled trials. BMJ Open 2014;4: e005983. doi:10.1136/ bmjopen-2014-005983
5. Joshi R, Alim M, Kengne AP, Jan S, Maulik P K, Peiries D Et al. Task shifting for non- communicable disease management in low middle income countries-a systematic review. PLoS ONE 9(8): e103754. doi: 10:137/journal pone.0103754
6. Callaghan M, Ford N, Schneider H. A systematic review of task shifting for HIV treatment and care in Africa. Hum Resour Health. 2010; 8:8–16
7. Atun R, Jaffar S, Nishtar S, et al. Improving responsiveness of health systems to non-communicable diseases. Lancet. 2013;381: 690–7
8. Moher D, Liberati A, Tetzlaff J, Altman DG, PRISMA Group (2009) Preferred reporting items for systematic reviews and meta-analyses: the PRISMA statement. Ann Intern Med 151: 264-269.
9. Fulton BD, Scheffler RM, Sparkes SP, Auh EY, Vujicic M, Soucat A. Health workforce skill mix and task shifting in low income countries: A review of recent evidence. Hum Resour Health 2011; 9:1. doi: 10.1186/1478-4491-9-1.
10. World Bank.2016 <https://datahelpdesk.worldbank.org/knowledgebase/articles/906519-world-bank-country-and-lending-groups>. Accessed on September 2016

**Table S1: Risk of bias Summary table for RCTs**

| Author | Random sequence  generation | Allocation concealment | Blinding of participants and personnel | Blinding of outcome assessment | Incomplete outcome data | Selective reporting | Others |
| --- | --- | --- | --- | --- | --- | --- | --- |
| Sartorelli | **?** | **?** | **+** | **?** | **+** | **?** | **?** |
| Jiang | **-** | **?** | **?** | **-** | **-** | **?** | **-** |
| Mollaoğlu | **?** | **?** | **?** | **?** | **?** | **?** | **?** |
| Andryukhin | **-** | **-** | **+** | **-** | **-** | **?** | **-** |
| Selvaraj | **-** | **-** | **+** | **?** | **-** | **-** | **-** |
| Saffi | **-** | **-** | **+** | **+** | **-** | **-** | **-** |
| Muchiri | **-** | **+** | **+** | **-** | **-** | **?** | **-** |
| Xavier | **-** | **-** | **?** | **+** | **-** | **-** | **?** |
| Ali | **-** | **-** | **+** | **+** | **-** | **-** | **-** |
| Zhang | - | - | + | - | - | ? | - |
| Mash^##^ | **-** | **?** | **+** | **+** | **+** | **-** | **-** |

^##^ *Additional Risk of bias for cRCT (Mash et al);Recruitment bias -low risk; Baseline imbalances- low risk-adjusted with statistical analysis; Incorrect analysis-low risk (stated considered for clustering); Comparability-Unclear risk*

**Table S2: Quality assessment for before and after studies (NHLBI Tool)**

| No |  | Pishdad | Denman | Navichraren | Kamran |
| --- | --- | --- | --- | --- | --- |
| 1 | Was the study question or objective clearly stated? | Yes | Yes | Yes | Yes |
| 2 | Were eligibility/selection criteria for the study population pre-specified and clearly described? | Yes | CD^#^ | Yes | Yes |
| 3 | Were the participants in the study representative of those who would be eligible for the test/service/intervention in the general or clinical population of interest? | No | No | No | Yes |
| 4 | Were all eligible participants that met the pre-specified entry criteria enrolled? | CD^#^ | CD^#^ | Yes | Yes |
| 5 | Was the sample size sufficiently large to provide confidence in the findings? | No | Yes | No | Yes |
| 6 | Was the test/service/intervention clearly described and delivered consistently across the study population? | Yes | Yes | Yes | Yes |
| 7 | Were the outcome measures pre-specified, clearly defined, valid, reliable, and assessed consistently across all study participants? | Yes | No | Yes | Yes |
| 8 | Were the people assessing the outcomes blinded to the participants' exposures/interventions? | No | No | No | No |
| 9 | Was the loss to follow-up after baseline 20% or less? Were those lost to follow-up accounted for in the analysis? | Yes | Yes | Yes | Yes |
| 10 | Did the statistical methods examine changes in outcome measures from before to after the intervention? Were statistical tests done that provided p values for the pre-to-post changes? | Yes | Yes | Yes | Yes |
| 11 | Were outcome measures of interest taken multiple times before the intervention and multiple times after the intervention (i.e., did they use an interrupted time-series design)? | NA | NA | NA | NA |
| 12 | If the intervention was conducted at a group level (e.g., a whole hospital, a community, etc.) did the statistical analysis take into account the use of individual-level data to determine effects at the group level? | NA | NA | NA | NA |

^#^CD- Cannot determine; NA- Not Applicable

**Table S3**: GRADE Evidence findings

| **Quality assessment** | | | | | | | **№ of patients** | | **Effect** | | **Quality** | **Importance** |
| --- | --- | --- | --- | --- | --- | --- | --- | --- | --- | --- | --- | --- |
| **№ of studies** | **Study design** | **Risk of bias** | **Inconsistency** | **Indirectness** | **Imprecision** | **Other considerations** | **Task shifting** | **usual care** | **Relative (95% CI)** | **Absolute (95% CI)** |  |  |
| Low Density Lipid (follow up: range 2 months to 24 months) | | | | | | | | | | | | |
| 8 | randomised trials | serious | serious | not serious | serious | all plausible residual confounding would suggest spurious effect, while no effect was observed | 1024 | 1010 | - | MD **6.90 mg/dl lower** (11.81 lower to 1.99 lower) | ⨁⨁◯◯ LOW | IMPORTANT |
| High Density Lipid (follow up: range 2 months to 12 months) | | | | | | | | | | | | |
| 7 | randomised trials | serious | not serious | not serious | serious | publication bias strongly suspected all plausible residual confounding would suggest spurious effect, while no effect was observed | 449 | 439 | - | MD **0.29 mg/dl higher** (1.12 lower to 1.94 higher) | ⨁⨁◯◯ LOW | NOT IMPORTANT |
| Total Cholesterol (follow up: range 2 months to 12 months) | | | | | | | | | | | | |
| 7 | randomised trials | serious ^a^ | serious ^b^ | not serious | serious ^c^ | all plausible residual confounding would suggest spurious effect, while no effect was observed | 449 | 439 | - | MD **9.44 mg/dl lower** (17.94 lower to 0.93 lower) | ⨁⨁◯◯ LOW | NOT IMPORTANT |
| Triglycerides (follow up: range 2 months to 12 months) | | | | | | | | | | | | |
| 4 | randomised trials | very serious ^d^ | serious ^e^ | not serious | very serious ^f^ | all plausible residual confounding would suggest spurious effect, while no effect was observed | 248 | 239 | - | MD **14.31 mg/dl lower** (33.32 lower to 4.69 higher) | ⨁◯◯◯ VERY LOW | NOT IMPORTANT |

**CI:** Confidence interval; **MD:** Mean difference

#### Explanations

a. High risk of bias

b. Wide variation in study population, intervention and task shifting strategies

c. Few study participants with wider CI

d. High risk of bias characterized by no Random Sequence Generation, Poor outcome assessment

e. Variations in interventions and study population

f. Fewer study participants with wide variation in features

**Table S4: PRISMA CHECKLIST**

| **Section/topic** | **#** | **Checklist item** | **Reported on page #** |
| --- | --- | --- | --- |
| **TITLE** | | |  |
| Title | 1 | Identify the report as a systematic review, meta-analysis, or both. | 1/Title |
| **ABSTRACT** | | |  |
| Structured summary | 2 | Provide a structured summary including, as applicable: background; objectives; data sources; study eligibility criteria, participants, and interventions; study appraisal and synthesis methods; results; limitations; conclusions and implications of key findings; systematic review registration number. | 2/Abstract |
| **INTRODUCTION** | | |  |
| Rationale | 3 | Describe the rationale for the review in the context of what is already known. | 3/Introduction |
| Objectives | 4 | Provide an explicit statement of questions being addressed with reference to participants, interventions, comparisons, outcomes, and study design (PICOS). | 3/Introduction |
| **METHODS** | | |  |
| Protocol and registration | 5 | Indicate if a review protocol exists, if and where it can be accessed (e.g., Web address), and, if available, provide registration information including registration number. | Supplementary file |
| Eligibility criteria | 6 | Specify study characteristics (e.g., PICOS, length of follow-up) and report characteristics (e.g., years considered, language, publication status) used as criteria for eligibility, giving rationale. | 4/Methods |
| Information sources | 7 | Describe all information sources (e.g., databases with dates of coverage, contact with study authors to identify additional studies) in the search and date last searched. | 4/Methods |
| Search | 8 | Present full electronic search strategy for at least one database, including any limits used, such that it could be repeated. | Supplementary file |
| Study selection | 9 | State the process for selecting studies (i.e., screening, eligibility, included in systematic review, and, if applicable, included in the meta-analysis). | 4/Methods |
| Data collection process | 10 | Describe method of data extraction from reports (e.g., piloted forms, independently, in duplicate) and any processes for obtaining and confirming data from investigators. | 5/Methods |
| Data items | 11 | List and define all variables for which data were sought (e.g., PICOS, funding sources) and any assumptions and simplifications made. | 5/Methods |
| Risk of bias in individual studies | 12 | Describe methods used for assessing risk of bias of individual studies (including specification of whether this was done at the study or outcome level), and how this information is to be used in any data synthesis. | 5/Methods |
| Summary measures | 13 | State the principal summary measures (e.g., risk ratio, difference in means). | 5/Methods |
| Synthesis of results | 14 | Describe the methods of handling data and combining results of studies, if done, including measures of consistency (e.g., I^2^) for each meta-analysis. | 5/Methods |

Page 1 of 2

| **Section/topic** | **#** | **Checklist item** | **Reported on page #** |
| --- | --- | --- | --- |
| Risk of bias across studies | 15 | Specify any assessment of risk of bias that may affect the cumulative evidence (e.g., publication bias, selective reporting within studies). | 5/Methods |
| Additional analyses | 16 | Describe methods of additional analyses (e.g., sensitivity or subgroup analyses, meta-regression), if done, indicating which were pre-specified. | 5/Methods |
| **RESULTS** | | |  |
| Study selection | 17 | Give numbers of studies screened, assessed for eligibility, and included in the review, with reasons for exclusions at each stage, ideally with a flow diagram. | 6/Results |
| Study characteristics | 18 | For each study, present characteristics for which data were extracted (e.g., study size, PICOS, follow-up period) and provide the citations. | 9/Results |
| Risk of bias within studies | 19 | Present data on risk of bias of each study and, if available, any outcome level assessment (see item 12). | 17/Results |
| Results of individual studies | 20 | For all outcomes considered (benefits or harms), present, for each study: (a) simple summary data for each intervention group (b) effect estimates and confidence intervals, ideally with a forest plot. | 9/Results |
| Synthesis of results | 21 | Present results of each meta-analysis done, including confidence intervals and measures of consistency. | 18/Results |
| Risk of bias across studies | 22 | Present results of any assessment of risk of bias across studies (see Item 15). | Results |
| Additional analysis | 23 | Give results of additional analyses, if done (e.g., sensitivity or subgroup analyses, meta-regression [see Item 16]). | Results and supplementary file |
| **DISCUSSION** | | |  |
| Summary of evidence | 24 | Summarize the main findings including the strength of evidence for each main outcome; consider their relevance to key groups (e.g., healthcare providers, users, and policy makers). | Discussion and conclusion |
| Limitations | 25 | Discuss limitations at study and outcome level (e.g., risk of bias), and at review-level (e.g., incomplete retrieval of identified research, reporting bias). | Discussion and conclusion |
| Conclusions | 26 | Provide a general interpretation of the results in the context of other evidence, and implications for future research. | Discussion and conclusion |
| **FUNDING** | | |  |
| Funding | 27 | Describe sources of funding for the systematic review and other support (e.g., supply of data); role of funders for the systematic review. | None |

*From:*  Moher D, Liberati A, Tetzlaff J, Altman DG, The PRISMA Group (2009). Preferred Reporting Items for Systematic Reviews and Meta-Analyses: The PRISMA Statement. PLoS Med 6(6): e1000097. doi:10.1371/journal.pmed1000097

For more information, visit: **www.prisma-statement.org**.

Page 2 of 2

Figure S1 : Funnel plot for LDL
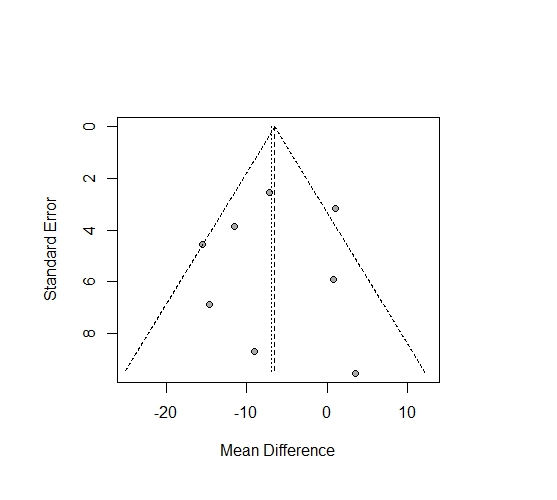


Figure S2: Forrest plot for effect of Task shifting interventions on LDL levels in different risk population
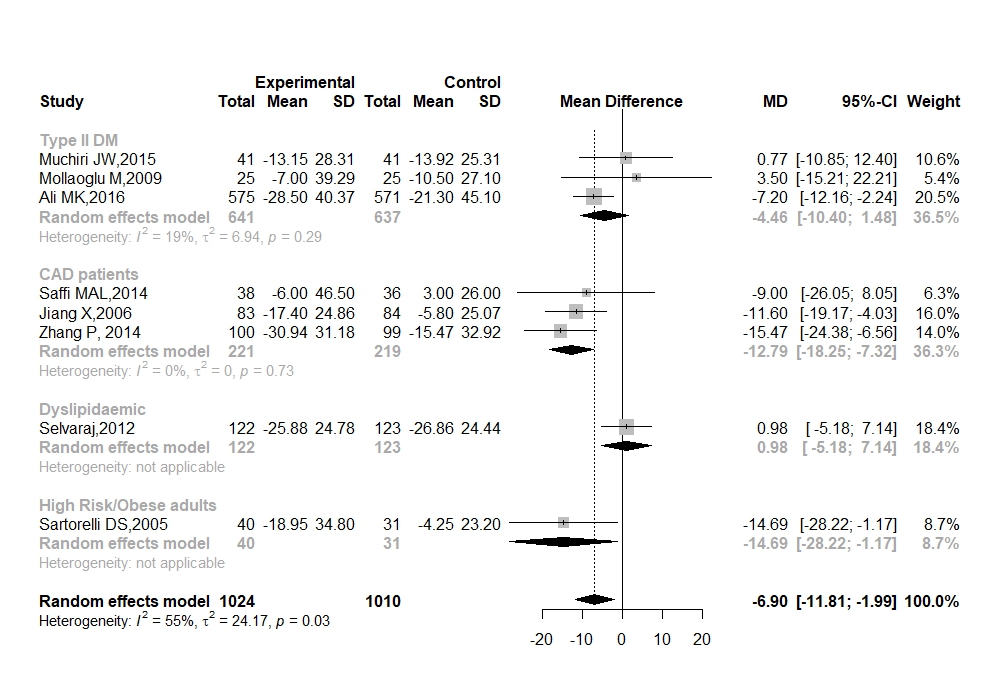


Figure S3: Forrest plot for effect of task sharing interventions on LDL levels using different task sharing strategy


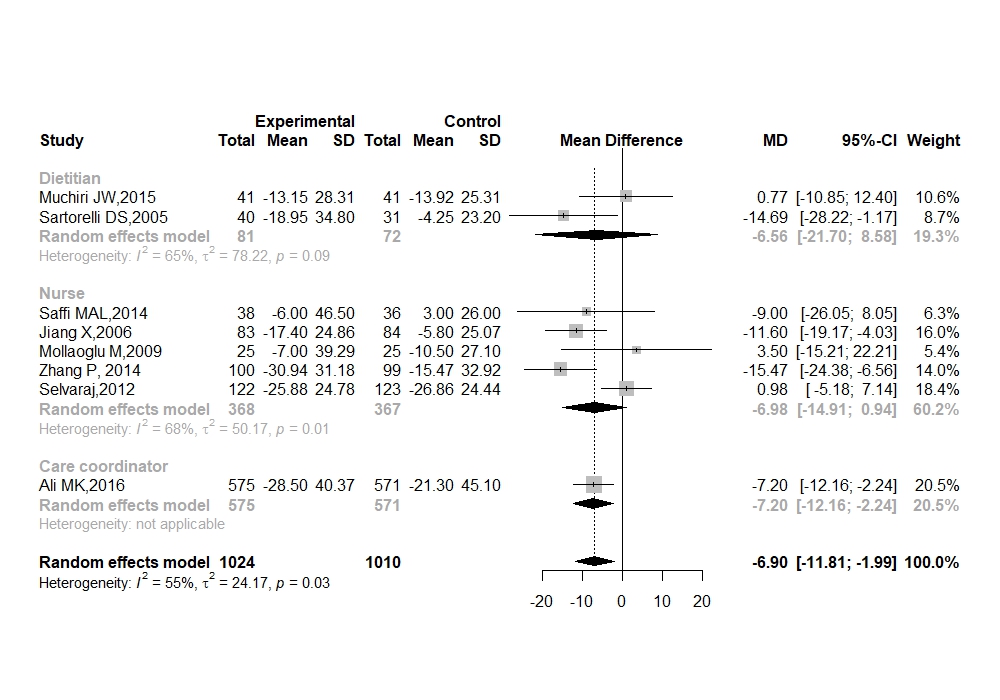


Figure S4: Funnel plot for HDL


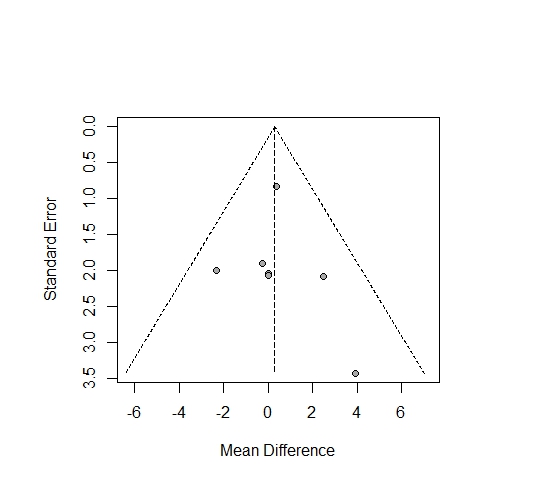


Figure S5: Forrest plot for effect of Task sharing interventions on HDL levels in different risk population


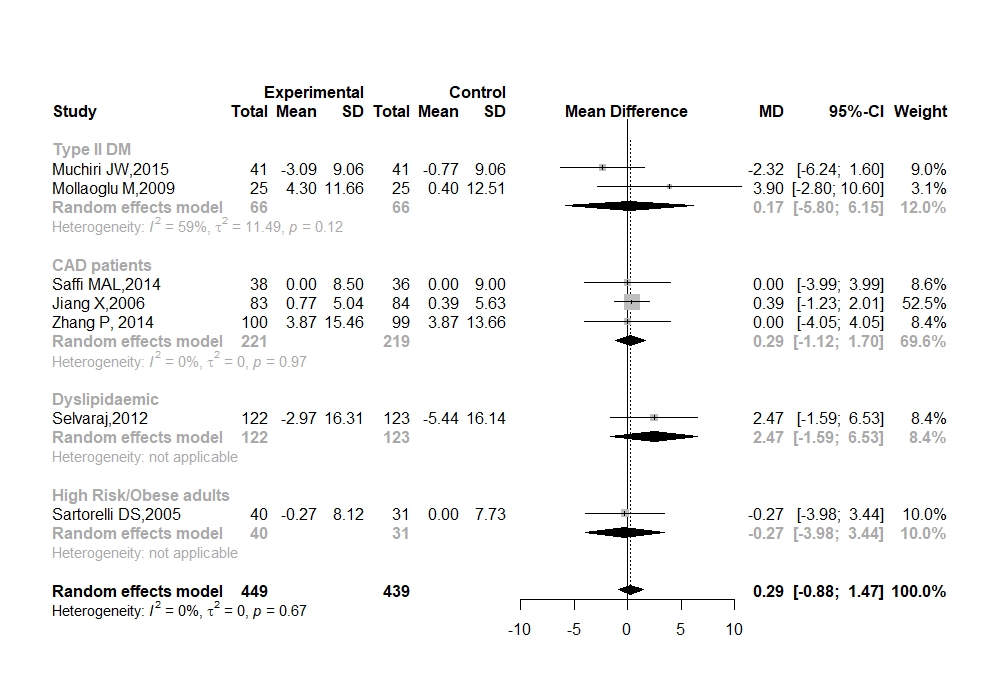


Figure S6: Forrest plot for effect of task sharing interventions on HDL levels using different task sharing strategy
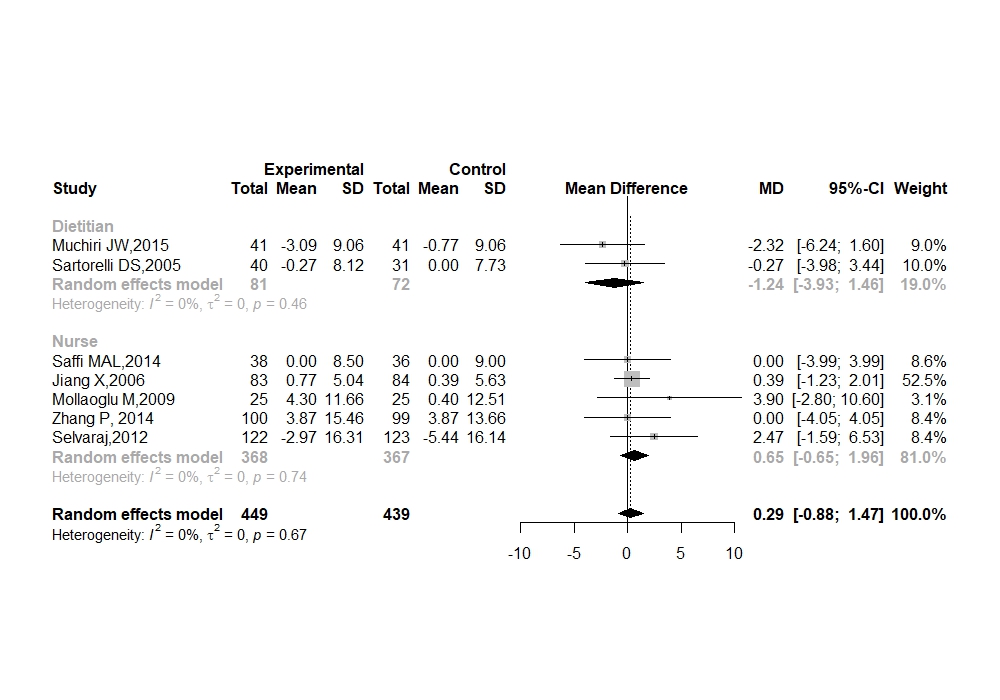


Figure S7: Funnel plot for Total Cholesterol (TC)


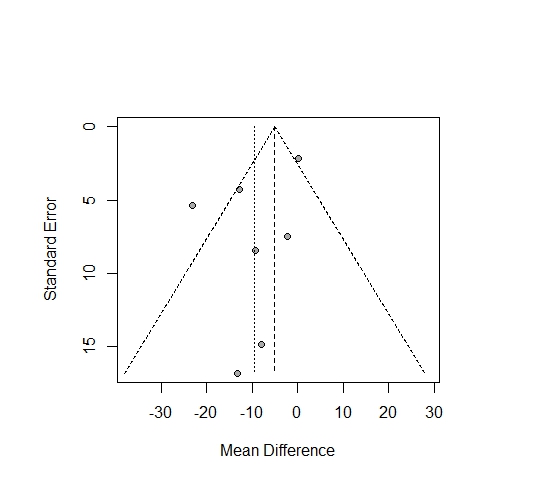


Figure S8: Forrest plot for effect of Task sharing interventions on TC levels in different risk population


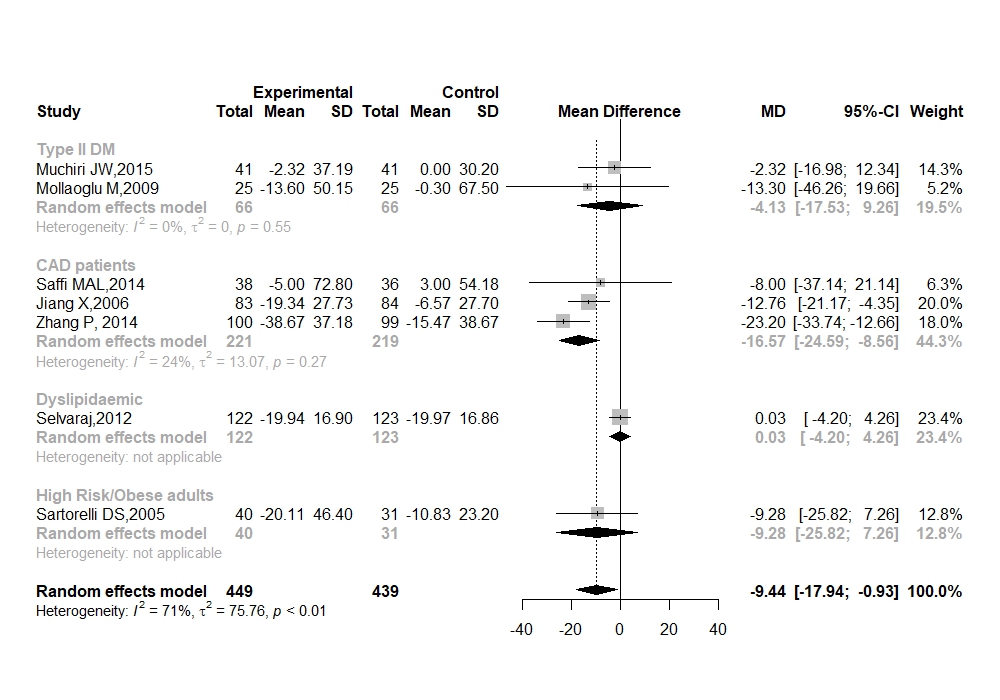


Figure S9 : Forrest plot for effect of Task sharing interventions on TC levels in levels using different task sharing strategy


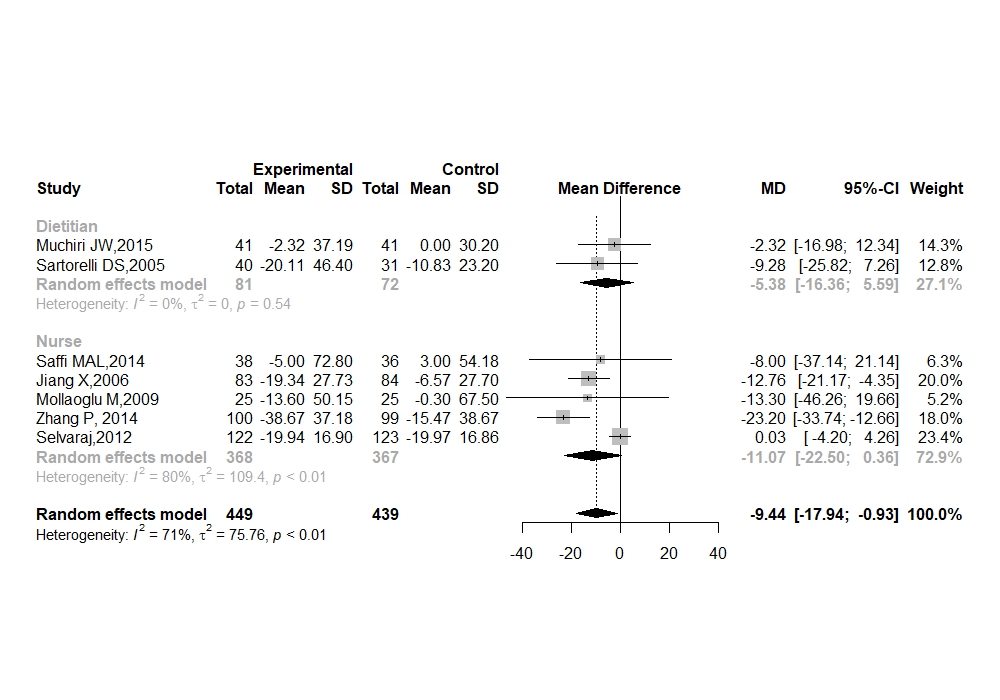


Figure S10: Funnel plot for Triglycerides (TG)
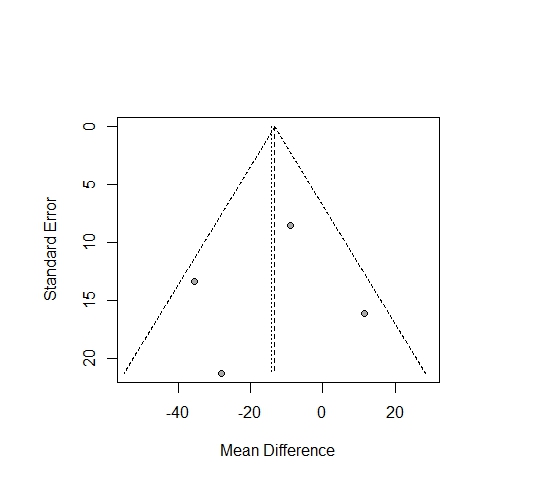


Figure S11: Forrest plot for effect of task sharing interventions on TG levels in different risk population


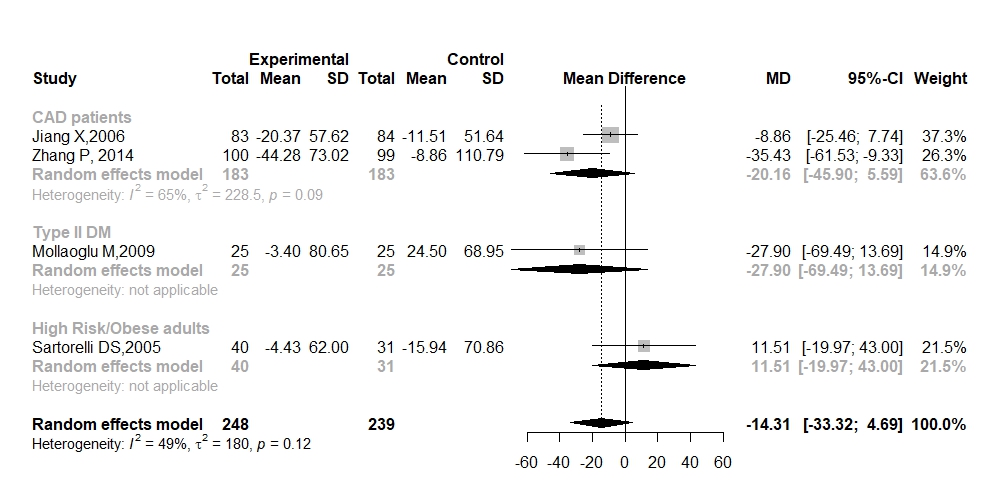


Figure S 12 : Forrest plot for effect of Task sharing interventions on TG levels in levels using different task sharing strategy


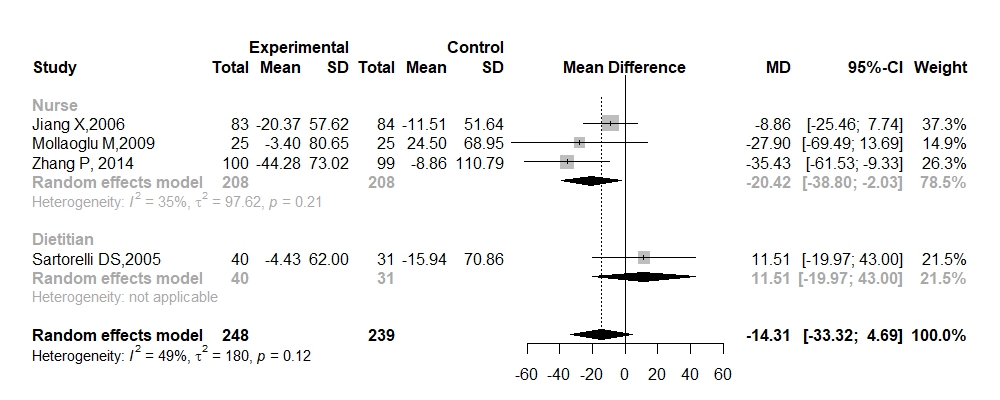

Supplement: Appendix [file mmc1.docx]
